# Comparative efficacy and safety of pharmacological treatments for lower-risk myelodysplastic syndromes: a Systematic Review and network meta-analysis

**DOI:** 10.3389/fonc.2026.1853205

**Published:** 2026-06-16

**Authors:** Haixia Zhang, Jiaqiang Hu, Yaxi Jiang, Lihua Deng, Song Liu

**Affiliations:** 1 Department of Pharmacy, Sichuan University West China Hospital Guang’an Hospital, Guang’an, Sichuan, China; 2 Department of Pharmacy, The Affiliated Hospital, Southwest Medical University, Luzhou, Sichuan, China

**Keywords:** erythroid response, lower-risk myelodysplastic syndromes, network meta-analysis, pharmacological treatment, transfusion independence

## Abstract

**Background:**

The comparative efficacy of pharmacological treatments for lower-risk myelodysplastic syndromes (LR-MDS) remains uncertain because head-to-head randomised evidence is limited.

**Methods:**

We performed a systematic review and network meta-analysis of randomised controlled trials evaluating pharmacological treatments for LR-MDS. Outcomes of interest were haematological improvement–erythroid (HI–E), red blood cell transfusion independence (RBC–TI), haematological improvement–platelet (HI–P), and adverse events (AEs).

**Results:**

We included 20 trials (2,877 participants), of which 17 trials (2,670 participants) contributed to connected treatment networks. For HI–E, luspatercept and ESA-based regimens were associated with higher rates. Lenalidomide-containing regimens showed favourable RBC–TI outcomes, including lenalidomide plus ESA, lenalidomide with or without ESA and/or G-CSF, and lenalidomide monotherapy. For HI–P, eltrombopag and romiplostim showed potential benefits. Safety profiles differed across treatments: CC-486 and imetelstat were associated with higher odds of neutropenia and gastrointestinal toxicity, whereas luspatercept was associated with increased fatigue and AEs.

**Conclusion:**

Comparative treatment benefit in LR-MDS differs by clinical endpoint. These findings support an individualised, goal-directed treatment approach rather than a single universally superior therapy.

**Systematic review registration:**

https://www.crd.york.ac.uk/prospero/, identifier CRD420251184384.

## 1 Introduction

Myelodysplastic syndromes (MDS) are a heterogeneous group of clonal haematopoietic disorders characterised by ineffective haematopoiesis and peripheral cytopenias, with a variable risk of progression to acute myeloid leukaemia (AML) (1, 2). In patients with lower-risk myelodysplastic syndromes (LR-MDS), the disease course is generally indolent. However, symptomatic anaemia is highly prevalent and strongly associated with fatigue, impaired quality of life, and frequent transfusion requirements (3).

Beyond transfusion support, multiple pharmacological strategies have been developed to address anaemia in LR-MDS. The therapeutic landscape has expanded from erythropoiesis-stimulating agents (ESAs) to agents targeting erythroid maturation (luspatercept), immunomodulators (lenalidomide), hypomethylation (azacitidine, decitabine), thrombopoietin receptor agonists (romiplostim, eltrombopag), and novel mechanisms (imetelstat, roxadustat) (4–9). However, these therapies target distinct biological pathways and demonstrate variable efficacy across haematological endpoints. Importantly, haematological improvement–erythroid (HI–E), red blood cell transfusion independence (RBC–TI), and haematological improvement–platelet (HI–P) are not interchangeable outcomes; therapies that improve erythroid parameters may not translate into durable transfusion independence (10, 11).

Despite this expanding therapeutic landscape, direct head-to-head comparisons across drug classes are scarce. Previous meta-analyses in LR-MDS focused on individual therapeutic classes such as ESAs, lenalidomide, luspatercept, or hypomethylating agents (12–15). Most were designed to confirm whether one treatment worked better than control, rather than to compare several active strategies within one framework. They also often focused on a single endpoint, usually erythroid response or transfusion outcomes, without separating the clinical meaning of HI–E, RBC–TI, and HI–P. Therefore, we conducted the first systematic review and network meta-analysis (NMA) to compare pharmacological strategies across HI–E, RBC–TI, and HI–P. Our aim was to provide an evidence-based framework to inform clinical decision-making and optimise therapeutic strategies, while accounting for differences in treatment efficacy and safety across clinically relevant outcomes.

## 2 Research design and methods

### 2.1 Protocol and registration

This systematic review and network meta-analysis was prospectively registered in PROSPERO (CRD420251184384) and conducted in accordance with PRISMA 2020 and PRISMA-NMA (16).

### 2.2 Data sources and search strategy

PubMed, Embase, the Cochrane Central Register of Controlled Trials (CENTRAL), and Web of Science were searched from database inception to October 9, 2025 for randomised controlled trials enrolling adults with LR-MDS. Additional eligible randomised trials identified in the updated search (February 23, 2026) could not be connected to the networks because of lacking a shared comparator. The complete search strategy is provided in **Appendix 1**.

### 2.3 Study selection and endpoints

Two reviewers (HX-Z and JQ-H) independently screened titles and abstracts and assessed full-text articles for eligibility. Disagreements were resolved through discussion and consensus, with adjudication by a third reviewer when necessary. We excluded 1) reviews, editorials, and letters without original data; 2) conference abstracts and non-English publications; 3) retrospective studies; and 4) studies enrolling patients with diseases other than LR-MDS or mixed populations without extractable data for the LR-MDS subgroup.

The primary efficacy endpoints were RBC–TI and HI–E, summarised with odds ratios (ORs) with 95% CI (Confidence Interval). For each included trial, we extracted the trial-defined primary endpoint according to International Working Group (IWG) response criteria. 19 of the 20 included trials used IWG-2006 criteria, whereas one trial (Mittelman2024) reported outcomes according to the revised IWG-2018 criteria (17). All analyses included only responses sustained for ≥8 weeks according to trial-defined criteria (18, 19).

### 2.4 Data extraction and risk of bias assessment

Data were independently extracted by two reviewers (HX-Z and JQ-H) using a standardised Excel spreadsheet developed specifically for this study. We collected key characteristics including the first author’s name, year of publication, clinical trial identifier, study design, and details of the study arms. Patient demographics, such as sample size, number and percentage of male participants, and age summary measures (mean or median, with dispersion measures as reported) were recorded when available. We also extracted information on baseline laboratory values such as haemoglobin levels, platelet counts, and serum erythropoietin levels.

We extracted efficacy outcomes, including HI–E, RBC–TI, and HI–P, along with the corresponding event counts and sample sizes for each study arm. We additionally collected International Prognostic Scoring System (IPSS) and revised International Prognostic Scoring System (IPSS-R), as well as information on RS status and World Health Organisation (WHO) classification. We extracted safety outcomes, including adverse events (AEs) such as fatigue, diarrhoea, nausea, dyspnoea, constipation, anaemia, and neutropenia, as well as serious adverse events (SAEs), according to the definitions reported in the original studies.

Risk of bias was assessed independently by two reviewers using the Cochrane Risk of Bias 2 (RoB 2) tool across the following domains: bias arising from the randomisation process, deviations from intended interventions, missing outcome data, measurement of the outcome, and selection of the reported result (20).

### 2.5 Statistical analysis

We performed random-effects network meta-analysis in a frequentist framework using R (v4.3.2) and the netmeta package. Treatment ranking was assessed using P-scores within the frequentist framework. P-scores reflect the mean extent of certainty that one treatment is superior to another based on estimated relative treatment effects and their precision (21). Dichotomous outcomes were summarised as ORs with 95% CIs. Additionally, heterogeneity was quantified using the *I*^2^ statistic and *Q* statistics for both within-design and between-design variability. To assess local inconsistency, we performed a node-splitting analysis, which separates evidence into direct and indirect components for each comparison.

### 2.6 Assessment of confidence in findings

We assessed the effect estimates for the primary outcomes using the Confidence In Network Meta-Analysis (CINeMA) framework and methodology (22, 23). CINeMA assesses within-study bias, reporting bias, indirectness, imprecision, heterogeneity, and incoherence, and summarises confidence. The final confidence rating for the evidence was determined by combining the results from all areas, resulting in one of four categories: very low, low, moderate, or high. The transitivity assumption was assessed by comparing the distribution of potential effect modifiers across treatment comparisons.

### 2.7 Sensitivity analysis

To assess the robustness of our findings, we performed two sensitivity analyses (1): Response criteria: We re-ran the RBC–TI network meta-analysis excluding the trial using IWG-2018 criteria (Mittelman2024) (2). Risk of bias: We re-ran HI–E and RBC–TI network meta-analyses excluding trials rated as high risk of bias in the RoB 2 assessment.

## 3 Results

### 3.1 Literature selection and study characteristics

**Figure 1:**
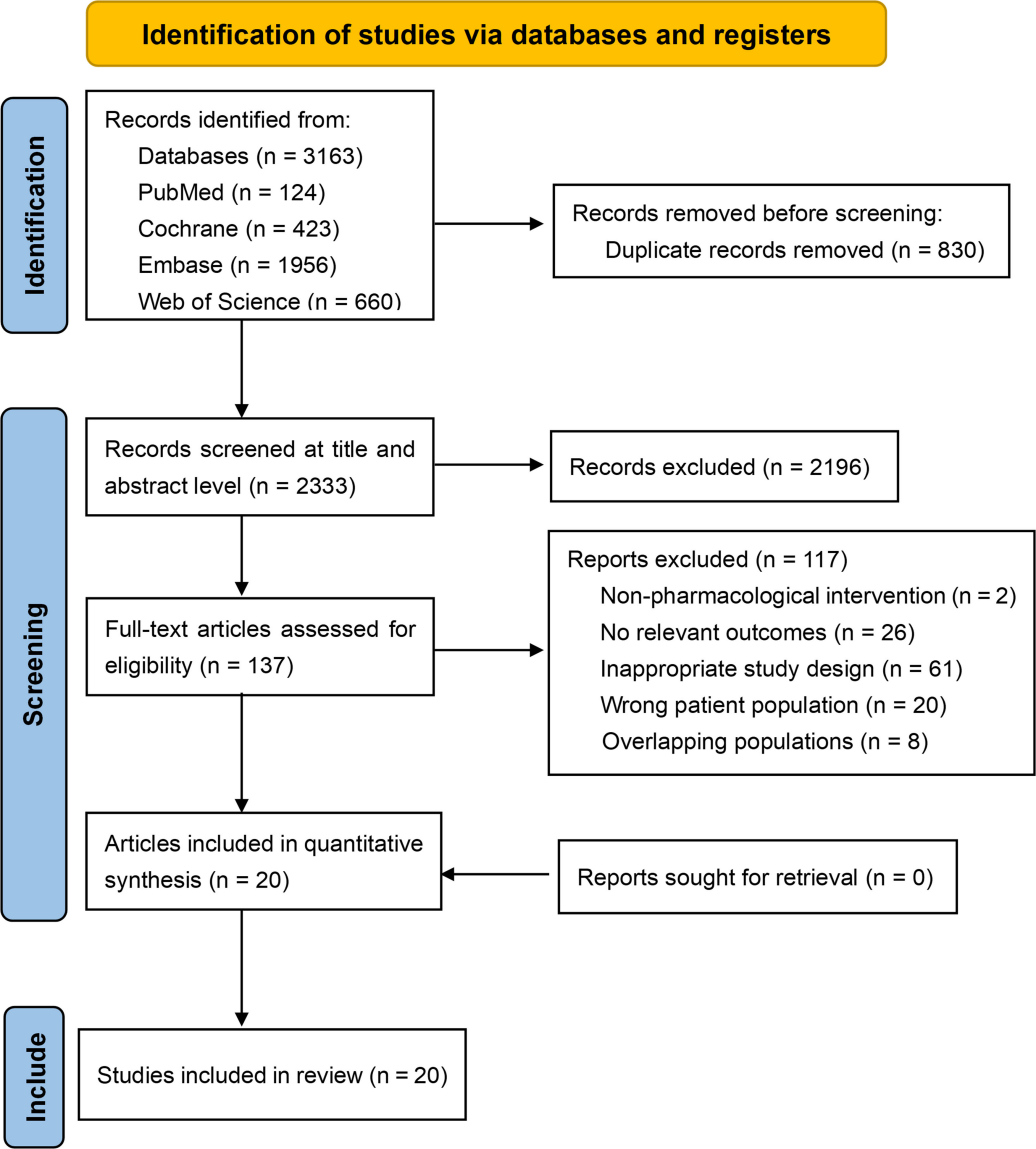
Flowchart of the study selection and exclusion criteria.

The systematic search identified 3,163 records across all databases, with 2,333 unique studies remaining after the removal of duplicates. After screening titles and abstracts, 2,196 studies were excluded, leaving 137 studies for full-text assessment. Of these, 117 were excluded for the following reasons: non-pharmacological intervention (n = 2), no relevant outcomes (n = 26), inappropriate study design (n = 61), wrong patient population (n = 20) and overlapping populations (n = 8). We included 20 randomised controlled trials (RCTs) (2,877 participants) in the systematic review. Among these trials, 17 trials (2,670 participants) contributed connected networks and were included in the network meta-analysis; the remaining trials could not be connected owing to the absence of a shared comparator and were synthesised narratively (**Figure 1**).

Among the 20 studies included in the analysis, the characteristics of the included studies was summarised in **Table 1** and the baseline patient demographics was reported in **Appendix 10**.

**Table 1:** Characteristics of the included studies.

Study	Phase	Unique identifier	Patients, n	Randomised treatments, n	Risk category (IPSS/IPSS-R) (%)	Outcomes
Balleari2006 (24)	–	–	30	ESA:15	L:40%; Int-1:60%	HI–E
				ESA + G-CSF:15	L:27%; Int-1:73%	
Toma2016 (25)	3	NCT01718379	131	Lenalidomide:65	L:46%; Int-1:55%	HI–E; RBC–TI
				Lenalidomide+ ESA:65	L:40%; Int-1:63%	
Thepot2016 (26)	2	NCT01015352	98	Azacitidine:49	L:39%; Int-1:59%; missing:2%	HI–E; RBC–TI
				Azacitidine + ESA:49	L:39%; Int-1:61%	
Platzbecker2017 (27)	3	NCT01362140	146	Placebo:49	L:51%; Int-1:49%	HI–E
				ESA:97	L:50%; Int-1:50%	
Fenaux2018 (28)	3	NCT01381809	130	Placebo:45	L:51%; Int-1:49%	HI–E
				ESA:85	L:41%; Int-1:58%; missing:1%	
Gattermann2018 (29)	2	NCT01868477	23	Deferasirox + ESA:11	–	HI–E; HI–P
				ESA:12	–	
Loosdrecht2024 (30)	2	EudraCT 2008-002195-10	184	Lenalidomide:92	L:46%; Int-1:54%	HI–E; RBC–TI
				Lenalidomide+/-ESA+/-G-CSF:92	L:38%; Int-1:60%; missing:2%	
Porta2024 (31)	3	NCT03682536	363	Luspatercept:182	VL:9%; L:71%; Int:19%; missing:1% ^a^	HI–E
				ESA:181	VL:9%; L:73%; Int:16%; missing:1% ^a^	
A.F. List2021 (32)	3	NCT00843882/NCT02048813	195	Lenalidomide + ESA:99	L:49%; Int-1:50%	HI–E
				Lenalidomide:96	L:38%; Int-1:62%	
Garcia-Manero2018 (33)	3	NCT01029262	239	Placebo:79	L:38%; Int-1:62%	HI–E; RBC–TI
				Lenalidomide:160	L:53%; Int-1:47%	
Garcia-Manero2021 (34)	3	NCT01566695	216	Placebo:109	Int-1:100%	HI–E; RBC–TI; HI–P
				CC-486:107	Int-1:100%	
Ye2021 (35)	2	CHiCTR-IPR-15006454	82	Placebo:41	L:7%; Int-1:88%	HI–P
				Decitabine:41	L:12%; Int-1:83%	
Fenaux2020 (36)	3	NCT02631070	229	Placebo:76	VL:8%; L:75%; Int:17% ^a^	HI–E
				Luspatercept:153	VL:12%; L:71%; Int:16% ^a^	
Platzbecker2024 (9)	3	NCT02598661	178	Placebo:60	L:65%; Int-1:35%	HI–E; RBC–TI
				Imetelstat:118	L:68%; Int-1:32%	
Mittelman2024 (17)	2/3	NCT03263091	140	Placebo:58	VL:12%; L:71%; Int:17% ^a^	RBC–TI ^b^
				Roxadustat:82	VL:7%; L:73%; Int:20% ^a^	
Giagounidis2014 (37)	2	NCT00614523	250	Placebo:83	L:28%; Int-1:70%; missing:2%	HI–P
				Romiplostim:167	L:24%; Int-1:72%; missing:4%	
Oliva2017 (38)	2	–	90	Placebo:31	L:42%; Int-1:58%	HI–P
				Eltrombopag:59	L:29%; Int-1:71%	
Schanz2009 (39)	2	–	44	Placebo:22	L:32%; Int-1:68%	HI–E; HI–P
				Amifostine:22	L:36%; Int-1:64%	
Ghaderi2020 (40)	2	–	27	β-D mannuronic acid + vitamin B12 + Folic acid:13	L:77%; Int-1:23%	HI–E; HI–P
				Vitamin B12 + Folic acid:14	L:86%; Int-1:14%	
Yang2020 (41)	–	ChiCTR-IOR-15006635	82	PND + Andriol Testocaps + Thalidomide:41	–	HI–P
				Andriol Testocaps + Thalidomide;41	–	

HI–E, haematological improvement–erythroid; HI–P, haematological improvement–platelet; RBC–TI, red blood cell transfusion independence; IPSS, International Prognostic Scoring System; VL, very low; L, low; Int-1, intermediate-1; IPSS-R, revised International Prognostic Scoring System; ESA, Erythropoiesis-stimulating agents.

^a^Patients were classified based on the IPSS-R.

^b^Response was assessed using the IWG-2018 criteria.

“–” = endpoint not assessed or not reported.

### 3.2 Risk of bias, certainty of evidence and consistency

Most trials were rated low risk or with some concerns, and 2 of 20 trials were rated high risk of bias (**Appendix 3**). For HI–E, between-study heterogeneity was low (τ² = 0.0235; I² = 5.3%), and we did not detect a global inconsistency signal (Q = 2.11; p = 0.3478) (**Appendix 2**). For RBC–TI and HI–P, heterogeneity and inconsistency statistics could not be reliably estimated because the networks were sparse. Comparison-adjusted funnel plots did not show asymmetry (**Appendix 4**). CINeMA ratings were generally low to moderate, most often downgraded due to imprecision and sparse direct comparisons (**Appendix 6**).

Quantitative assessment of effect modifier distributions identified substantial imbalances across treatment nodes (**Appendix 12**). Baseline EPO differed by 86.5 mU/mL between ESA trials (median 73.5 mU/mL) and lenalidomide trials (160 mU/mL). RS-positive prevalence was 73% in luspatercept trials versus 62% in imetelstat trials; RS status was not reported in lenalidomide trials but is estimated at 30-40% in unselected populations. These imbalances indicate high risk of transitivity violation for ESA-lenalidomide and luspatercept-lenalidomide comparisons.

Node-splitting analyses did not identify evidence of important local inconsistency in the available loops for HI–E, RBC–TI, HI–P, or AEs. However, the limited number of head-to-head trials reduces power to detect incoherence (**Appendix 8**).

### 3.3 Sensitivity analysis

Detailed endpoint definitions for each included trial are provided in **Appendix 11**. Although IWG-2006 and IWG-2018 differ in minimum response duration, both share an ≥8-week threshold as the primary RBC–TI and HI–E criterion. All 20 trials reported responses with ≥8-week duration, ensuring conceptual comparability across the network.

Excluding the IWG-2018 trial (Mittelman2024) from the RBC–TI network yielded unchanged effect estimates and treatment rankings. After excluding high risk of bias trials, effect estimates remained stable for HI–E and RBC–TI, and treatment rankings were preserved (**Appendix 9**).

### 3.4 Network meta-analysis of endpoint

#### 3.4.1 HI–E

**Figure 2:**
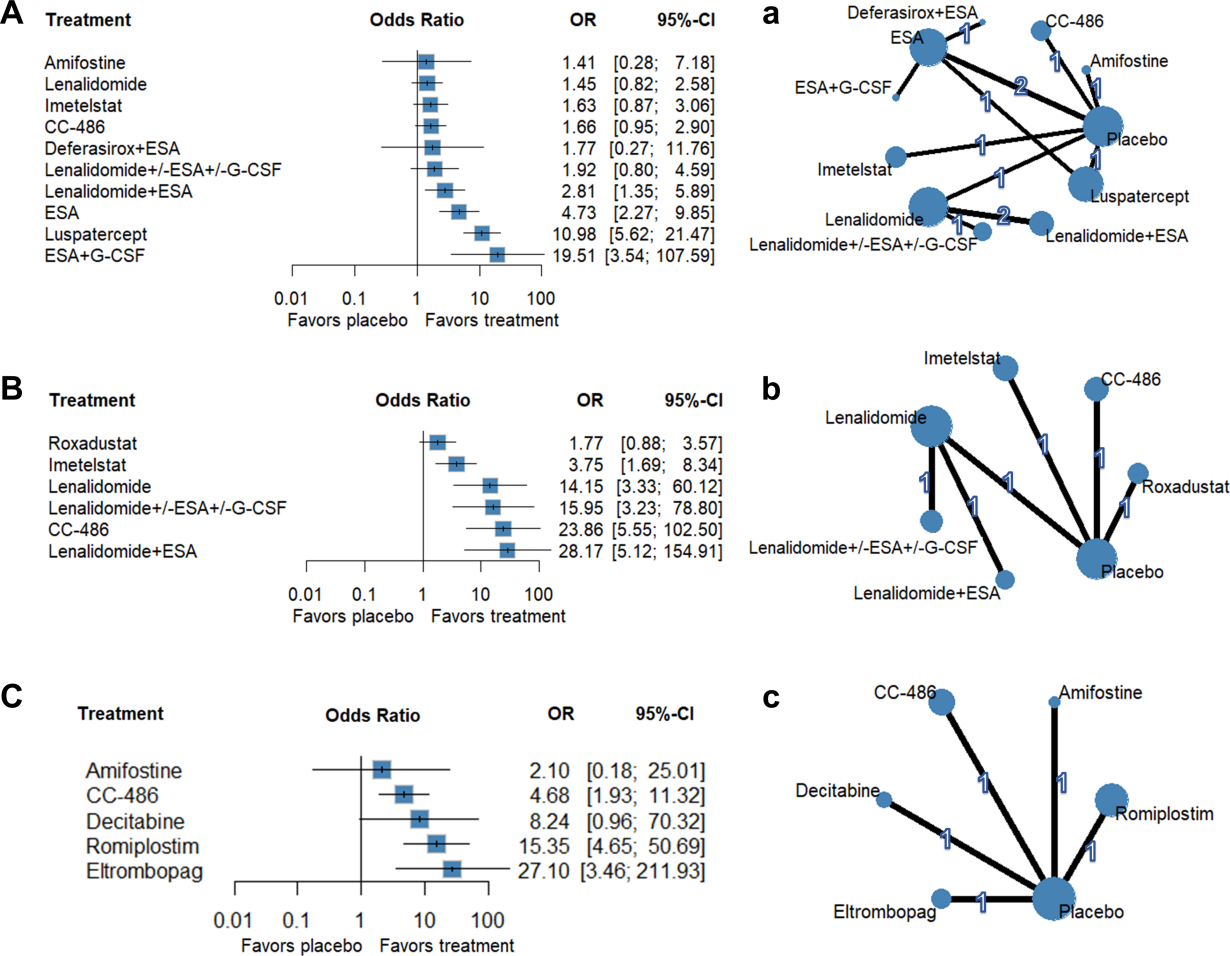
Network plots and forest plots for efficacy outcomes in LR-MDS. Node size is proportional to the number of participants, and line thickness reflects the number of participants contributing to direct head-to-head comparisons. **(A-a)** HI–E; **(B-b)** RBC–TI; **(C-c)** HI–P.

As displayed in **Figure 2**, 13 randomised trials comprising 2,108 patients contributed to the HI–E network. ESA plus granulocyte colony-stimulating factor (G-CSF) (OR 19.51, CI 3.54 – 107.59; P-score: 0.96) showed the largest effect estimate. Compared with placebo, luspatercept was associated with a marked increase in the odds of achieving HI–E and ranked second in efficacy (OR 10.98, 95% CI 5.62 – 21.47; P-score: 0.92). ESA monotherapy (OR 4.73, 95% CI 2.27 – 9.85; P-score: 0.75) yielded higher odds of achieving HI–E, followed by the combination of lenalidomide plus ESA (OR 2.81, 95% CI 1.35 – 5.89; P-score: 0.62).

Lenalidomide with or without ESA and/or G-CSF (Lenalidomide+/-ESA+/-G-CSF) (OR 1.92, 95% CI 0.80 – 4.59; P-score: 0.43), ESA plus deferasirox (OR 1.77, 95% CI 0.27 – 11.76; P-score: 0.39), oral azacitidine (CC-486; OR 1.66, 95% CI 0.95 – 2.90; P-score: 0.37), imetelstat (OR 1.63, 95% CI 0.87 – 3.06; P-score: 0.36), lenalidomide (OR 1.45, 95% CI 0.82 – 2.58; P-score: 0.28) and amifostine (OR 1.41, 95% CI 0.28 – 7.18; P-score: 0.31) did not reach statistical significance (**Figures 2A-a**; **Appendix 7**).

Using a pooled placebo event rate of 23.4% for HI−E, luspatercept corresponded to an absolute risk increase of 53.6% (95% CI 34.5% – 63.3%; NNT 1.9), with narrower CIs than those for RBC−TI and HI−P (**Appendix 13**).

#### 3.4.2 RBC–TI

Six trials enrolling 1,088 patients informed the RBC–TI network. Lenalidomide-containing strategies yielded the largest effect estimates versus placebo, although with substantial imprecision: lenalidomide plus ESA (OR 28.17, 95% CI 5.12 – 154.91; P-score: 0.89), lenalidomide+/-ESA+/-G-CSF (OR 15.95, 95% CI 3.23 – 78.80; P-score: 0.68), and lenalidomide monotherapy (OR 14.15, 95% CI 3.33 – 60.12; P-score: 0.61). Imetelstat (OR 3.75, 95% CI 1.69 – 8.34; P-score: 0.34) and CC-486 (OR 3.60, 95% CI 1.74 – 7.46; P-score: 0.79) were also more effective than placebo.

In contrast, roxadustat did not demonstrate a statistically significant improvement in RBC–TI compared with placebo (OR 1.77, 95% CI 0.88 – 3.57; P-score: 0.17). These estimates were imprecise, reflected by wide CIs. According to P-score based ranking, lenalidomide-containing regimens were most likely to rank among the top-performing strategies for achieving RBC–TI (**Figures 2B-b**; **Appendix 7**).

Translating these odds ratios to absolute effects using the pooled placebo event rate (10.5%), lenalidomide plus ESA corresponded to an absolute risk increase of 74.0% (95% CI 34.3% – 95.1%; NNT 1.4, 95% CI 1.1 – 2.9), whereas imetelstat showed a more modest absolute benefit of 22.4% (95% CI 6.5% – 43.0%; NNT 4.5, 95% CI 2.3–15.4) (**Appendix 13**).

#### 3.4.3 HI–P

Five trials including 682 patients contributed to the HI–P network. Eltrombopag was associated with the largest point estimate favouring platelet response (OR 27.10, 95% CI 3.46 – 211.93; P-score: 0.87). Romiplostim was associated with a statistically significant increase in the odds of platelet response compared with placebo and ranked second (OR 15.35, 95% CI 4.65 – 50.69; P-score: 0.77).

Among the LR-MDS agents, CC-486 (OR 4.68, 95% CI 1.93 – 11.32; P-score: 0.43) demonstrated more moderate but statistically significant effects, whereas decitabine (OR 8.24, 95% CI 0.96 – 70.32; P-score: 0.60) and amifostine (OR 2.10, 95% CI 0.18 – 25.01; P-score: 0.27) failed to show a significant benefit (**Figures 2C-c**; Appendix 7). Detailed pairwise network estimates for these efficacy outcomes are shown in **Appendix 5**.

Using the pooled placebo event rate (4.5%), eltrombopag corresponded to an absolute risk increase of 51.9% (95% CI 9.5% – 86.4%; NNT 1.9, 95% CI 1.2 – 10.5) (**Appendix 13**).

#### 3.4.4 Safety analysis

To facilitate clinical interpretation, safety outcomes were analysed in two categories: haematological toxicities (neutropenia, anaemia) and non-haematological toxicities (dyspnoea, fatigue, gastrointestinal events), in addition to composite outcomes (any AEs, SAEs, death).

**Figure 3:**
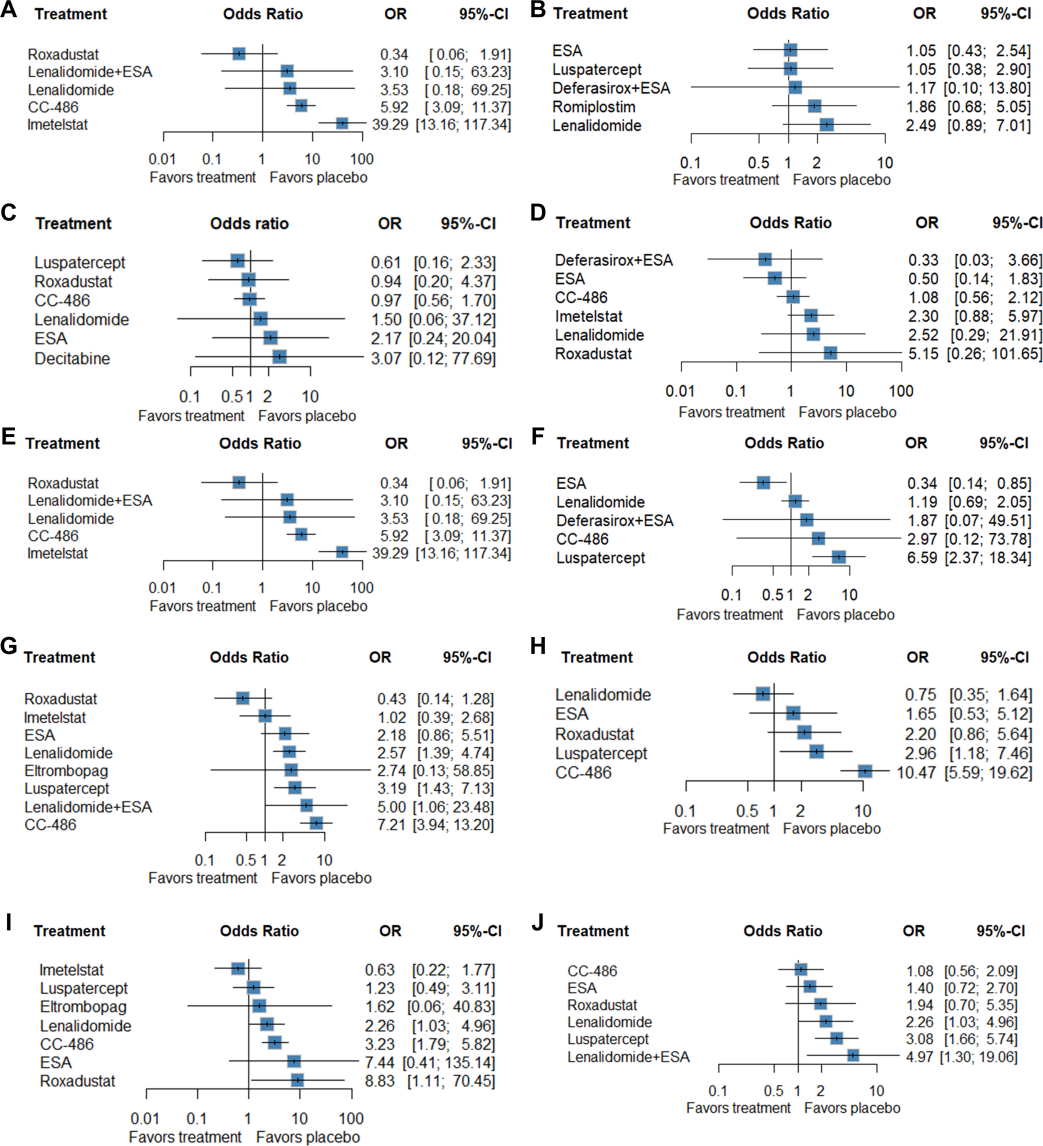
Network meta-analysis estimates for adverse events. Adverse events are grouped as follows: composite outcomes **(A–C)**, haematologic toxicities **(D, E)**, and non-haematologic toxicities **(F–J)**. **(A)** AEs; **(B)** SAEs; **(C)** Death; **(D)** Anaemia; **(E)** Neutropenia; **(F)** Dyspnoea; **(G)** Diarrhoea; **(H)** Nausea; **(I)** Constipation; **(J)** Fatigue.

**Figure 4:**
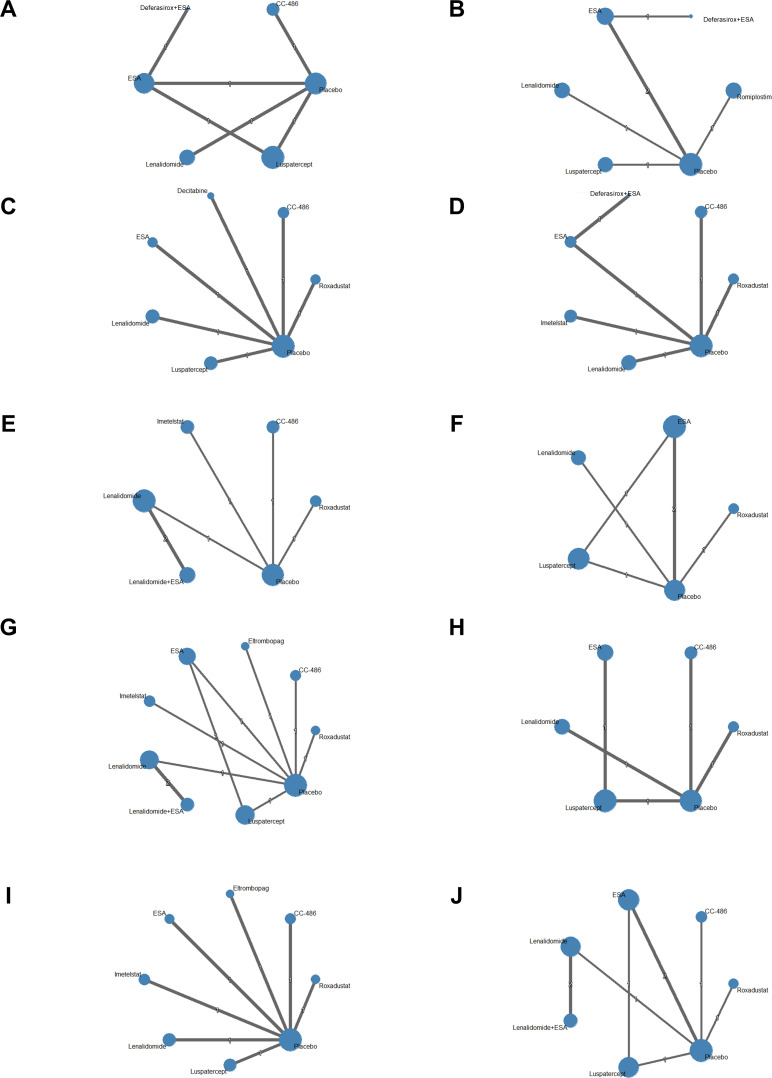
Network plots for adverse event comparisons in LR-MDS. Adverse events are grouped as follows: composite outcomes **(A–C)**, haematologic toxicities **(D, E)**, and non-haematologic toxicities **(F–J)**. **(A)** AEs; **(B)** SAEs; **(C)** Death; **(D)** Anaemia; **(E)** Neutropenia; **(F)** Dyspnoea; **(G)** Diarrhoea; **(H)** Nausea; **(I)** Constipation; **(J)** Fatigue.

As shown in **Figures 3**, **4**, the results revealed that luspatercept was associated with a higher incidence of AEs (OR: 6.59, 95% CI: 2.37 – 18.34, P-score: 0.11) and dyspnoea events (OR: 3.70, 95% CI: 1.37 – 9.99, P-score: 0.24). No therapy showed a clear difference versus placebo for SAEs or death, although power was limited for rare events (**Appendix 7**).

Haematological toxicities were prominent for neutropenia, particularly with imetelstat (OR 39.29, 95% CI 13.16–117.34; P-score: 0.03) and CC-486 (OR 5.92, 95% CI 3.09 – 11.37; P-score: 0.34) (**Figures 3E**, **4E**; **Appendix 7**). Regarding anaemia events, all evaluated therapies failed to show statistically significant differences (**Figures 3D**, **4D**; **Appendix 7**).

Among non-haematological AEs, gastrointestinal toxicity was most frequently reported. For diarrhoea events, CC-486 (OR 7.21, 95% CI 3.94 – 13.20; P-score: 0.09), luspatercept (OR 3.19, 95% CI 1.43 – 7.13; P-score: 0.32) and lenalidomide (OR 2.57, 95% CI 1.39 – 4.74; P-score: 0.43) were associated with increased odds (**Figures 3G**, **4G**; **Appendix 7**). CC-486 (OR 10.47, 95% CI 5.59 – 19.62; P-score: 0.003) and luspatercept (OR 2.96, 95% CI 1.18 – 7.46; P-score: 0.28) were also associated with a higher risk of nausea (**Figures 3H**, **4H**; **Appendix 7**). In terms of constipation, roxadustat (OR 8.83, 95% CI 1.11 – 70.45; P-score: 0.15), CC-486 (OR 3.23, 95% CI 1.79 – 5.82; P-score: 0.31) and lenalidomide (OR 2.26, 95% CI 1.03 – 4.96; P-score: 0.44) were associated with higher odds of constipation (**Figures 3I**, **4I**; **Appendix 7**). Lenalidomide plus ESA (OR 4.97, 95% CI 1.30 – 19.06; P-score: 0.09) and luspatercept (OR 3.08, 95% CI 1.66 – 5.74; P-score: 0.21) demonstrated higher odds of fatigue (**Figures 3J**, **4J**; Appendix 7). Detailed network meta-analysis results for safety outcomes are presented in **Appendix 5**.

### 3.5 Supplementary evidence from non-network comparisons

Several randomised trials could not be incorporated into the primary network due to the absence of a shared comparator. These studies were synthesised descriptively to provide additional context.

For HI–E, 2 trials (n = 125) provided direct active-comparator evidence. In a randomised trial comparing azacitidine monotherapy with azacitidine plus ESA, HI–E rates were 30.6% and 26.5%, respectively (OR 0.82, 95% CI 0.34 – 1.97). A study evaluating β-D mannuronic acid combined with vitamin B12 and folic acid reported a higher HI–E rate than vitamins alone (OR 18.76, 95% CI 0.92 – 383.5). For RBC–TI, a randomised trial comparing azacitidine with azacitidine plus ESA showed no evidence of benefit from combination therapy (OR 0.85, 95% CI 0.28 – 2.57).

3 trials including 132 patients contributed to the endpoint of HI–P. A small trial evaluating β-D mannuronic acid in combination with vitamins did not demonstrate a statistically significant improvement after continuity correction (OR 3.40, 95% CI 0.13 – 91.6). An additional trial (n = 82) evaluated a traditional Chinese medicine-integrated regimen. The combination of PND (a ginseng and astragalus-based herbal supplement), Andriol Testocaps (testosterone undecanoate), and thalidomide resulted in a significantly higher HI–P response rate (51.2%) compared to Andriol Testocaps plus thalidomide alone (26.8%) (OR 2.86, 95% CI 1.14 – 7.19). Another small trial directly compared the combination of deferasirox plus ESA versus ESA monotherapy in patients with LR-MDS. The HI–P response rates were 4 of 11 patients (36.4%) in the deferasirox plus ESA arm and 6 of 12 patients (50.0%) in the ESA monotherapy arm (OR 0.57, 95% CI 0.11 – 3.04). It indicated no statistically significant difference between the two regimens for improving HI–P.

### 3.6 Subgroup and meta-regression analyses

Planned subgroup analyses by RS status and transfusion dependence were not feasible due to insufficient data: RS status was reported in only 3/20 treatment nodes with no trials providing RS-stratified outcomes; all RBC–TI trials enrolled 100% transfusion-dependent patients, eliminating variation for this modifier.

Trial-level meta-regression (n=4 trials) showed baseline EPO was positively associated with HI–E effect size (β=0.58 per 100 U/L, 95% CI 0.13-1.03; p=0.012). This ecological finding is hypothesis-generating only given the small sample and potential confounding.

Descriptive comparisons showed luspatercept (73% RS-positive) had larger HI–E effects (OR 2.54) than imetelstat (62% RS-positive, OR 1.63), consistent with known biology, though confounded by other trial-level differences.

## 4 Discussion

In this systematic review and network meta-analysis, we compared the efficacy and safety of pharmacological treatments for LR-MDS across 3 clinically relevant haematological endpoints: HI–E, RBC–TI, and HI–P. The principal finding is that treatment effects were endpoint-specific. No single therapy was consistently superior across all outcomes. This is clinically important because these endpoints reflect different aspects of disease burden and are not interchangeable measures of benefit.

For HI–E, luspatercept and ESA-based strategies ranked highly. This pattern is biologically plausible and consistent with the known mechanisms of these agents. ESAs primarily amplify early erythropoiesis and are most effective when the serum erythropoietin level is low and marrow responsiveness is preserved (2, 42). Luspatercept acts later in erythroid maturation by trapping select ligands of the TGF-β superfamily and corrects ineffective late-stage maturation that is prominent in MDS with ring sideroblasts (RS) (1, 43). The strong HI–E signal seen with luspatercept is therefore consistent with both its mechanism and the characteristics of the trial population (36).

RBC–TI is a more demanding endpoint than haemoglobin rise alone. It reflects not only erythroid response, but also its depth, durability, and clinical impact in relation to baseline transfusion burden (44, 45). Lenalidomide has established activity in transfusion-dependent LR-MDS, particularly in patients with del(5q), where responses can be deep and durable (4). It may also improve transfusion outcomes in selected patients without del(5q), especially when combined with ESA support. However, the RBC–TI network was small, and several estimates were imprecise. For this reason, our results should not be interpreted as showing that lenalidomide-based therapy is the preferred option for all patients in LR-MDS. Rather, they suggest that these regimens may be especially relevant when RBC–TI is the main treatment goal and the biological setting is appropriate.

For HI–P, thrombopoietin receptor agonists had the most favourable effect estimates. Eltrombopag ranked highest, followed by romiplostim. This is in line with their lineage-directed mechanism of action and supports a practical point: treatment choice in LR-MDS should be linked to the dominant cytopenia. Agents that improve erythroid outcomes are not expected to have the same impact on platelet response, and the reverse is also true. Our results therefore support a goal-directed approach rather than a single hierarchy of best treatment.

The safety network should be interpreted as hypothesis-generating comparative signal detection rather than a definitive toxicity ranking, given between-trial variability in AE definitions, ascertainment intensity, and reporting practices. CC-486 and imetelstat were associated with higher risks of neutropenia and gastrointestinal toxicity, reflecting on-target marrow suppression and systemic exposure. Lenalidomide and luspatercept more often produced constitutional symptoms and treatment-emergent AEs that can affect adherence. These trade-offs support individualised selection based on the dominant clinical problem, baseline comorbidity profile, and tolerance. This approach is consistent with contemporary guideline frameworks that stratify therapy by transfusion burden, serum erythropoietin level, cytogenetics such as del(5q), and the presence of RS (46).

Previous meta-analyses in LR-MDS have primarily focused on single therapeutic classes or individual endpoints. Alhajahjeh et al. (13) synthesised evidence for luspatercept, while Wan and Han (12) evaluated hypomethylating agents specifically for transfusion independence. Park et al. (15) assessed erythropoiesis-stimulating agents in isolation, and Liu et al. (47) compared azacitidine and decitabine without including other drug classes. These studies typically evaluated a single primary endpoint without distinguishing between clinically distinct outcomes such as HI–E, RBC–TI, and HI–P. Unlike prior meta-analyses that focused on single drug classes or endpoints, our NMA simultaneously compares multiple strategies across HI–E, RBC–TI, and HI–P.

Our findings extend prior meta-analysis evidence in three ways. First, we provide the first comprehensive network comparison across 16 drug classes, enabling indirect comparisons that were previously unavailable. Second, we separately analyse clinically distinct endpoints revealing that treatment rankings differ substantially depending on the therapeutic goal. In our analysis, lenalidomide-containing regimens showed the largest relative effect estimates for RBC–TI, while luspatercept and ESA-based approaches were more prominent for HI–E. In practice, the treatment that improves anaemia-related response is not always the one most likely to achieve durable transfusion freedom. This endpoint-specific variation was not captured in prior single-endpoint meta-analyses. Third, we incorporate the most recent trial evidence, including studies of roxadustat and imetelstat, providing an up-to-date synthesis of the evolving therapeutic landscape. These incremental contributions support a goal-directed treatment approach rather than a single hierarchy of efficacy.

At the same time, our network highlights an issue that is less visible in earlier meta-analyses: biological enrichment across trials may influence indirect comparisons. Lenalidomide studies often included patients with del(5q) or marked transfusion dependence. Luspatercept trials were enriched for RS-positive disease. ESA studies tended to include patients with lower serum erythropoietin levels. These differences are clinically appropriate, but they also limit the extent to which one can interpret treatment rankings as universal across all LR-MDS populations.

This study has several limitations. First, CINeMA assessment rated most comparisons as low to moderate certainty, primarily downgraded for imprecision and sparse direct evidence. Several large odds ratios in this network meta-analysis were accompanied by extremely wide confidence intervals. When converted to absolute effects, the uncertainty remained substantial. Moreover, several high−ranking treatments were tested in single small trials, raising the possibility of small−study effects that may inflate effect estimates. Therefore, P−scores and relative rankings should not be overinterpreted as definitive evidence of superiority; they are best viewed as hypothesis−generating. Second, substantial data gaps precluded subgroup network meta-analyses. Quantitative assessment identified high-risk transitivity violations for two key comparisons (1): ESA versus lenalidomide, confounded by 86.5 mU/mL difference in baseline EPO, a known predictor of ESA response (2); luspatercept versus lenalidomide, confounded by >23% difference in RS prevalence, a known predictor of luspatercept response. Consequently, treatment rankings are conditional on trial populations and may not generalise to patients with different biomarker profiles. Luspatercept’s high P-score (0.92) for HI–E reflects efficacy in RS-positive patients and likely overestimates benefit in RS-negative populations. Additionally, safety outcomes were not uniformly defined across trials, and rare events were underpowered. Heterogeneous AE reporting across trials introduced systematic uncertainty. Therefore, our safety comparisons are hypothesis-generating and should not be overinterpreted as definitive risk hierarchies. Finally, the exploratory ecological meta-regression on serum EPO included only a small number of trials and should be considered hypothesis-generating only.

Despite these limitations, the study has practical implications. It supports a treatment strategy based on the dominant clinical goal rather than a single overall ranking. Our findings are broadly consistent with prior class-specific evidence. Low certainty evidence suggests that lenalidomide−containing regimens may improve RBC−TI, whereas moderate certainty evidence supports luspatercept and ESA−based strategies for HI−E. If the main goal is HI–E, luspatercept and ESA-based approaches appear most relevant. If the goal is durable RBC–TI, lenalidomide-containing regimens may be more important in selected patients. If thrombocytopenia is the main issue, thrombopoietin receptor agonists appear most active. In routine practice, treatment choice should still be guided by disease biology, prior therapy, transfusion burden, safety profile, and patient preference.

To aid real−world treatment selection, our results can be aligned with NCCN/ESMO guidelines as follows: in non−del(5q) LR−MDS with anaemia and low EPO, ESA−based therapy is a reasonable first step. Among ESA−refractory patients, RS positivity favours luspatercept for HI−E, while transfusion dependence with no clear RS or del(5q) may shift consideration to lenalidomide−containing regimens or, where available, imetelstat/CC−486 with awareness of myelosuppression. For del(5q) patients, lenalidomide remains the benchmark. For thrombocytopenia, TPO−RAs represent a signal−driven option. These suggestions are exploratory, given the low−to−moderate certainty of evidence, and should be tailored to individual comorbidities and patient preference (**Appendix 14**).

Future research should prioritise head-to-head trials that directly test the top candidates within a defined therapeutic goal. Trials should also integrate patient-reported outcomes and standardised transfusion metrics to link haematological responses to quality of life. Finally, biomarker-driven strategies will be essential. Prospective validation of predictors such as serum erythropoietin, RS or SF3B1 status, and molecular profiles may enable more precise sequencing and move LR-MDS therapy from goal-directed care towards true precision medicine.

## 5 Conclusion

In this network meta-analysis of randomised trials in LR-MDS, therapeutic efficacy differed substantially across endpoints, including HI–E, RBC–TI, and HI–P. Luspatercept and ESA-based strategies showed the most favourable results for HI–E, whereas lenalidomide-containing regimens were associated with the greatest likelihood of achieving RBC–TI. Thrombopoietin receptor agonists showed the most favourable results for HI–P. However, most comparisons were supported by low to moderate certainty evidence, with sensitivity analyses confirming the robustness of HI–E and RBC–TI rankings. Treatment rankings should be interpreted as hypothesis-generating in light of the certainty of evidence, and clinical decisions should integrate effect estimates, certainty ratings, and safety profiles. In clinical practice, treatment selection in LR-MDS should therefore be guided by the dominant cytopenia, transfusion burden, and molecular profile, rather than by a single ranking of efficacy across all endpoints.

## Data Availability

The original contributions presented in the study are included in the article/**Supplementary Material**. Further inquiries can be directed to the corresponding author.
