# Supplementary material for: Comparative efficacy and safety of pharmacological treatments for lower-risk myelodysplastic syndromes: a Systematic Review and network meta-analysis
Source: Front Oncol. 2026 Jun 16;16:1853205. doi: 10.3389/fonc.2026.1853205 (PMC13314470; doi:10.3389/fonc.2026.1853205)
Supplement: Supplementary file 1 [file Table1.docx]

**Comparative Efficacy and Safety of Pharmacological Treatments for Lower-Risk Myelodysplastic Syndromes: A Systematic Review and Network Meta-analysis**

[Appendix 1. Search strategy of the study 3](#_Toc229338679)

[Table 1.1 PubMed search strategy 3](#_Toc229338680)

[Table 1.2 Web of Science search strategy 3](#_Toc229338681)

[Table 1.3 Cochrane search strategy 4](#_Toc229338682)

[Table 1.4 Embase search strategy 4](#_Toc229338683)

[Appendix 2. Evaluation of heterogeneity and inconsistency 5](#_Toc229338684)

[Table 2.1 Evaluation of heterogeneity and inconsistency for efficacy outcomes 5](#_Toc229338685)

[Table 2.2 Evaluation of heterogeneity and inconsistency for safety outcomes 5](#_Toc229338686)

[Appendix 3. Risk of bias assessment 6](#_Toc229338687)

[Table 3.1 Risk of bias assessment of included trials for HI–E 6](#_Toc229338688)

[Table 3.2 Risk of bias assessment of included trials for RBC–TI 7](#_Toc229338689)

[Table 3.3 Risk of bias assessment of included trials for HI–P 7](#_Toc229338690)

[Table 3.4 Risk of bias assessment of included trials for AEs 8](#_Toc229338691)

[Appendix 4. Comparison-adjusted funnel plots for HI–E 10](#_Toc229338692)

[10](#_Toc229338693)

[Appendix 5. Network Meta-analysis results 11](#_Toc229338694)

[Table 5.1 Network Meta-analysis results for HI–E 11](#_Toc229338695)

[Table 5.2 Network Meta-analysis results for RBC–TI 13](#_Toc229338696)

[Table 5.3 Network Meta-analysis results for HI–P 14](#_Toc229338697)

[Table 5.4 Network Meta-analysis results for AEs 15](#_Toc229338698)

[Table 5.5 Network Meta-analysis results for Dyspnoea 16](#_Toc229338699)

[Table 5.6 Network Meta-analysis results for SAEs 17](#_Toc229338700)

[Table 5.7 Network Meta-analysis results for Death 18](#_Toc229338701)

[Table 5.8 Network Meta-analysis results for Neutropenia 19](#_Toc229338702)

[Table 5.9 Network Meta-analysis results for Anaemia 20](#_Toc229338703)

[Table 5.10 Network Meta-analysis results for Diarrhoea 21](#_Toc229338704)

[Table 5.11 Network Meta-analysis results for Nausea 22](#_Toc229338705)

[Table 5.12 Network Meta-analysis results for Constipation 23](#_Toc229338706)

[Table 5.13 Network Meta-analysis results for Fatigue 24](#_Toc229338707)

[Appendix 6. Certainty of the effect estimates 25](#_Toc229338708)

[Table 6.1 Certainty of the effect estimates for HI–E 25](#_Toc229338709)

[Table 6.2. Certainty of the effect estimates for RBC–TI 29](#_Toc229338710)

[Table 6.3. Certainty of the effect estimates for HI–P 30](#_Toc229338711)

[Table 6.4. Certainty of the effect estimates for AEs 31](#_Toc229338712)

[Appendix 7. Treatment ranking (P-scores) 33](#_Toc229338713)

[Table 7.1 Treatment ranking (P-scores) for efficacy outcomes 33](#_Toc229338714)

[Table 7.2 Treatment ranking (P-scores) for safety outcomes 34](#_Toc229338715)

[Appendix 8. Forest plot of node-splitting analysis 36](#_Toc229338716)

[Figure 8.1 Forest plot of node-splitting analysis for HI–E 36](#_Toc229338717)

[Figure 8.2 Forest plot of node-splitting analysis for HI–P 37](#_Toc229338718)

[Figure 8.3 Forest plot of node-splitting analysis for AEs 38](#_Toc229338719)

[Appendix 9. Sensitivity analysis 39](#_Toc229338720)

[Table 9.1. Sensitivity analysis for RBC–TI after excluding the IWG-2018 Trial 39](#_Toc229338721)

[Table 9.2. Sensitivity analysis for HI–E after excluding high risk of bias trials 39](#_Toc229338722)

[Table 9.3. Sensitivity analysis for RBC–TI after excluding high risk of bias trials 40](#_Toc229338723)

[Appendix 10. Baseline patient characteristics 41](#_Toc229338724)

[Appendix 11. Summary of endpoint definitions and response criteria 46](#_Toc229338725)

[Appendix 12. Baseline characteristics and effect modifier distribution across treatment nodes 49](#_Toc229338726)

[Appendix 13. Absolute risk differences and number needed to treat 51](#_Toc229338727)

[Appendix 14. Hypothesis‑generating clinical framework for treatment selection in LR‑MDS 52](#_Toc229338728)

## Appendix 1. Search strategy of the study

### Table 1.1 PubMed search strategy

| 7 | (((lower-risk[Title/Abstract]) OR (lower risk[Title/Abstract])) AND ((“Myelodysplastic Syndromes”[Mesh]) OR (((Myelodysplastic Syndrome[Title/Abstract]) OR (Syndrome*, Dysmyelopoietic[Title/Abstract])) OR (Myelodysplasia*, Hematopoetic[Title/Abstract])))) AND ((((“Randomized Controlled Trials as Topic”[Mesh] OR “Randomized Controlled Trial” [Publication Type]) OR (“Controlled Clinical Trial” [Publication Type] OR “Controlled Clinical Trials as Topic”[Mesh])) OR (“Randomized Controlled Trials as Topic”[Mesh] OR “Randomized Controlled Trial” [Publication Type] OR “Controlled Clinical Trial” [Publication Type] OR “Controlled Clinical Trials as Topic”[Mesh])) OR ((clinical trial[Text Word]) OR (randomly[Text Word]))) |
| --- | --- |
| 6 | (((“Randomized Controlled Trials as Topic”[Mesh] OR “Randomized Controlled Trial” [Publication Type]) OR (“Controlled Clinical Trial” [Publication Type] OR “Controlled Clinical Trials as Topic”[Mesh])) OR (“Randomized Controlled Trials as Topic”[Mesh] OR “Randomized Controlled Trial” [Publication Type] OR “Controlled Clinical Trial” [Publication Type] OR “Controlled Clinical Trials as Topic”[Mesh])) OR ((clinical trial[Text Word]) OR (randomly[Text Word])) |
| 5 | ((lower-risk[Title/Abstract]) OR (lower risk[Title/Abstract])) AND ((“Myelodysplastic Syndromes”[Mesh]) OR (((Myelodysplastic Syndrome[Title/Abstract]) OR (Syndrome*, Dysmyelopoietic[Title/Abstract])) OR (Myelodysplasia*, Hematopoetic[Title/Abstract]))) |
| 4 | (lower-risk[Title/Abstract]) OR (lower risk[Title/Abstract]) |
| 3 | (“Myelodysplastic Syndromes”[Mesh]) OR (((Myelodysplastic Syndrome[Title/Abstract]) OR (Syndrome*, Dysmyelopoietic[Title/Abstract])) OR (Myelodysplasia*, Hematopoetic[Title/Abstract])) |
| 2 | ((Myelodysplastic Syndrome[Title/Abstract]) OR (Syndrome*, Dysmyelopoietic[Title/Abstract])) OR (Myelodysplasia*, Hematopoetic[Title/Abstract]) |
| 1 | “Myelodysplastic Syndromes”[Mesh] |

### Table 1.2 Web of Science search strategy

| 1 | (((TS=(Myelodysplastic Syndromes)) OR TS=(Myelodysplastic Syndrome)) OR TS=(Syndrome*, Dysmyelopoietic)) OR TS=(Myelodysplasia*, Hematopoetic) |
| --- | --- |
| 2 | (TS=(lower-risk)) OR TS=(lower risk) |
| 3 | #2 AND #1 |
| 4 | ((((TS=(Randomized Controlled Trial*)) OR TS=(controlled clinical tria*)) OR TS=(randomly)) OR TS=(clinical tria*)) OR TS=(placeb*) |
| 5 | #4 AND #3 |

### Table 1.3 Cochrane search strategy

| #1 | (randomized controlled tria*):ti,ab,kw OR (controlled clinical tria*):ti,ab,kw OR (randomly):ti,ab,kw OR (clinical tria*):ti,ab,kw OR (placeb*):ti,ab,kw (Word variations have been searched) |
| --- | --- |
| #2 | MeSH descriptor: [Myelodysplastic Syndromes] explode all trees |
| #3 | (Myelodysplastic Syndrome*):ti,ab,kw OR (Syndrome*, Dysmyelopoietic):ti,ab,kw OR (Myelodysplasia*, Hematopoetic):ti,ab,kw (Word variations have been searched) |
| #4 | (lower-risk):ti,ab,kw OR (lower risk):ti,ab,kw (Word variations have been searched) |
| #5 | #2 OR #3 |
| #6 | #4 AND #5 |
| #7 | #1 AND #6 |

### Table 1.4 Embase search strategy

| #5 | #3 AND #4 |
| --- | --- |
| #4 | ‘randomized controlled trial’/exp OR ‘randomized controlled trial’ OR (randomized AND controlled AND (‘trial’/exp OR trial)) OR ‘randomized controlled tria*’:ti,ab,kw OR ‘lower risk’:ti,ab,kw OR ‘controlled clinical tria*’:ti,ab,kw OR ‘clinical tria*’:ti,ab,kw OR randomly:ti,ab,kw |
| #3 | #1 AND #2 |
| #2 | ‘lower risk’ OR ‘lower risk’:ti,ab,kw |
| #1 | ‘myelodysplastic syndromes’/exp OR ‘myelodysplastic syndromes’ OR (myelodysplastic AND syndromes) OR ‘myelodysplastic syndrome’:ti,ab,kw OR ‘syndrome*, dysmyelopoietic’:ti,ab,kw OR ‘myelodysplasia*, hematopoetic’:ti,ab,kw |

## Appendix 2. Evaluation of heterogeneity and inconsistency

### Table 2.1 Evaluation of heterogeneity and inconsistency for efficacy outcomes

| Outcome | Estimated τ^2^ | I^2^ | Q | p-value |
| --- | --- | --- | --- | --- |
| HI–E | 0.0235 | 5.3% | 2.11 | 0.3478 |
| RBC–TI | NA | NA | NA | NA |
| HI–P | NA | NA | NA | NA |

NA indicates that heterogeneity statistics were not estimable because the corresponding network contained insufficient closed loops.

### Table 2.2 Evaluation of heterogeneity and inconsistency for safety outcomes

| Outcome | Estimated τ^2^ | I^2^ | Q | p-value |
| --- | --- | --- | --- | --- |
| AEs | 0.2833 | 36.1% | 3.13 | 0.2090 |
| SAEs | 0.1739 | 42.8% | 1.75 | 0.1862 |
| Death | NA | NA | NA | NA |
| Anaemia | NA | NA | NA | NA |
| Dyspnoea | 0.1137 | 18.2% | 2.44 | 0.2947 |
| Neutropenia | 0 | 0 | 0.05 | 0.8249 |
| Diarrhoea | 0 | 0 | 0.92 | 0.8195 |
| Constipation | 0 | 0 | 0 | 0.9852 |

NA indicates that heterogeneity statistics were not estimable because the corresponding network contained insufficient closed loops.

## Appendix 3. Risk of bias assessment

### Table 3.1 Risk of bias assessment of included trials for HI–E

| Study ID | Randomisation process | Deviations from intended interventions | Missing outcome data | Measurement of the outcome | Selection of the reported result | Overall Bias |
| --- | --- | --- | --- | --- | --- | --- |
| Fenaux2018 | Low | Low | Low | Low | High | Some concerns |
| Gatterman2018 | Low | Some concerns | Low | Low | Low | Some concerns |
| A.F. List 2021 | Low | Low | Low | Low | Low | Low |
| Schanz 2009 | Low | Some concerns | Low | Low | Low | Some concerns |
| Toma2016 | Low | Some concerns | Low | Low | High | High |
| Loosdrecht2024 | Low | Low | Low | Low | High | Some concerns |
| Garcia-Manero2018 | Low | Low | Low | Low | High | High |
| Platzbecker2017 | Low | Low | Low | Low | High | Some concerns |
| Garcia-Manero 2021 | Low | Low | Low | Low | Low | Low |
| Platzbecker2024 | Low | Low | Low | Low | Low | Low |
| Fenaux2020 | Low | Low | Low | Low | Low | Low |
| Porta2024 | Low | Low | Low | Low | Low | Low |
| Balleari2006 | Low | Some concerns | Low | Low | Low | Some concerns |

### Table 3.2 Risk of bias assessment of included trials for RBC–TI

| Study ID | Randomisation process | Deviations from intended interventions | Missing outcome data | Measurement of the outcome | Selection of the reported result | Overall Bias |
| --- | --- | --- | --- | --- | --- | --- |
| Toma2016 | Low | Some concerns | Low | Low | High | High |
| Loosdrecht2024 | Low | Low | Low | Low | High | Some concerns |
| Garcia-Manero2018 | Low | Low | Low | Low | High | High |
| Garcia-Manero 2021 | Low | Low | Low | Low | Low | Low |
| Platzbecker2024 | Low | Low | Low | Low | Low | Low |
| Mittelman2024 | Low | Low | Low | Low | High | Some concerns |

### Table 3.3 Risk of bias assessment of included trials for HI–P

| Study ID | Randomisation process | Deviations from intended interventions | Missing outcome data | Measurement of the outcome | Selection of the reported result | Overall Bias |
| --- | --- | --- | --- | --- | --- | --- |
| Schanz 2009 | Low | Some concerns | Low | Low | Low | Some concerns |
| Garcia-Manero 2021 | Low | Low | Low | Low | Low | Low |
| Oliva2017 | Low | Low | Low | Low | Low | Low |
| Ye2021 | Low | Low | Low | Low | Low | Low |
| Giagounidis2014 | Low | Low | Low | Low | Low | Low |

### Table 3.4 Risk of bias assessment of included trials for AEs

| Study ID | Randomisation process | Deviations from intended interventions | Missing outcome data | Measurement of the outcome | Selection of the reported result | Overall Bias |
| --- | --- | --- | --- | --- | --- | --- |
| Fenaux2018 | Low | Low | Low | Low | High | Some concerns |
| Gatterman2018 | Low | Some concerns | Low | Low | Low | Some concerns |
| A.F. List 2021 | Low | Low | Low | Low | Low | Low |
| Schanz 2009 | Low | Some concerns | Low | Low | Low | Some concerns |
| Toma2016 | Low | Some concerns | Low | Low | High | High |
| Loosdrecht2024 | Low | Low | Low | Low | High | Some concerns |
| Garcia-Manero2018 | Low | Low | Low | Low | High | High |
| Platzbecker2017 | Low | Low | Low | Low | High | Some concerns |
| Garcia-Manero 2021 | Low | Low | Low | Low | Low | Low |
| Platzbecker2024 | Low | Low | Low | Low | Low | Low |
| Fenaux2020 | Low | Low | Low | Low | Low | Low |
| Mittelman2024 | Low | Low | Low | Low | High | Some concerns |
| Porta2024 | Low | Low | Low | Low | Low | Low |
| Oliva2017 | Low | Low | Low | Low | Low | Low |
| Ye2021 | Low | Low | Low | Low | Low | Low |
| Balleari2006 | Low | Some concerns | Low | Low | Low | Some concerns |
| Giagounidis2014 | Low | Low | Low | Low | Low | Low |

## Appendix 4. Comparison-adjusted funnel plots for HI–E

**
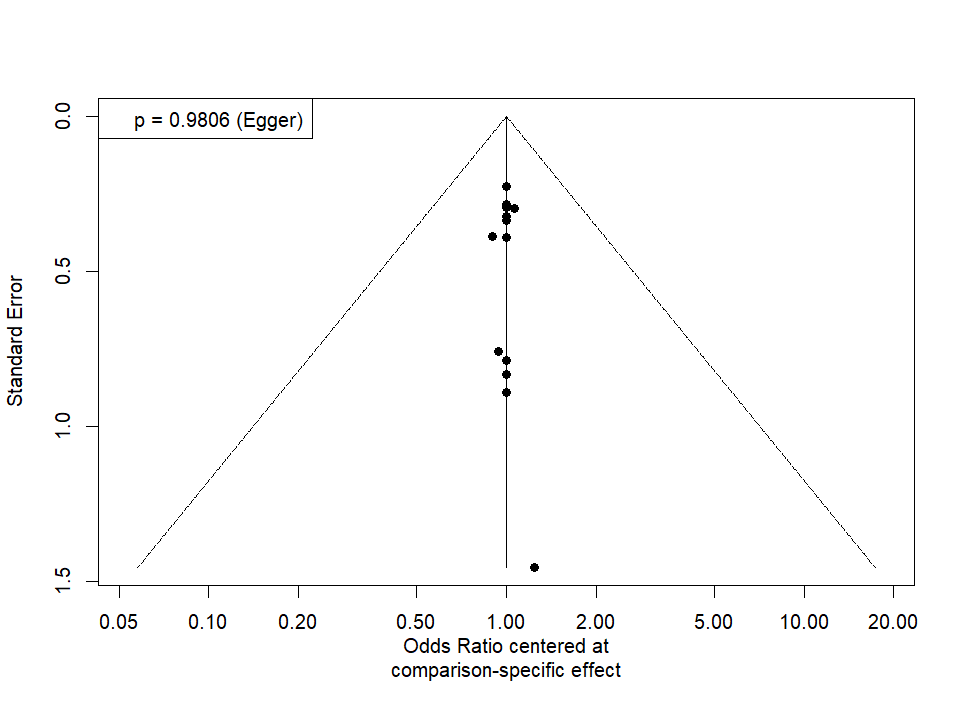
**

## Appendix 5. Network Meta-analysis results

### Table 5.1 Network Meta-analysis results for HI–E

The columns represent the comparison of the row drug class to the column drug class. The rows represent the comparison of the row drug class to the column drug class. The effect estimates are expressed as odds ratio and 95% confidence interval. Odds ratio <1 favors the drug in the column, Odds ratio >1 favors the drug in the row.

| Amifostine | . | . | . | . | . | . | . | . | . |  |
| --- | --- | --- | --- | --- | --- | --- | --- | --- | --- | --- |
| 0.97 (0.17 – 5.47) | Lenalidomide | . | . | . |  |  | . | . | . |  |
| 0.86 (0.15 – 4.95) | 0.89 (0.38 – 2.09) | Imetelstat | . | . | . | . | . | . | . |  |
| 0.85 (0.15 – 4.74) | 0.87 (0.39 – 1.94) | 0.98 (0.42 – 2.27) | CC-486 | . | . | . | . | . | . |  |
| 0.79 (0.07 – 9.64) | 0.82 (0.11 – 5.91) | 0.92 (0.13 – 6.76) | 0.94 (0.13 – 6.74) | Deferasirox + ESA | . | . |  | . | . | . |
| 0.73 (0.12 – 4.67) | 0.76 (0.39 – 1.46) | 0.85 (0.29 – 2.50) | 0.87 (0.31 – 2.45) | 0.93 (0.12 – 7.44) | Lenalidomide+/- ESA +/- G-CSF | . | . | . | . | . |
| 0.50 (0.08 – 2.99) | 0.52 (0.32 – 0.82) | 0.58 (0.22 – 1.53) | 0.59 (0.23 – 1.49) | 0.63 (0.08 – 4.80) | 0.68 (0.31 – 1.52) | Lenalidomide + ESA | . | . | . | . |
| 0.30 (0.05 – 1.78) | 0.31 (0.12 – 0.78) | 0.35 (0.13 – 0.91) | 0.35 (0.14 – 0.88) | 0.37 (0.07 – 2.14) | 0.41 (0.13 – 1.27) | 0.60 (0.21 – 1.69) | ESA |  |  |  |
| 0.13 (0.02 – 0.75) | 0.13 (0.05 – 0.32) | 0.15 (0.06 – 0.37) | 0.15 (0.06 – 0.36) | 0.16 (0.03 – 0.97) | 0.17 (0.06 – 0.52) | 0.26 (0.09 – 0.69) | 0.43 (0.28 – 0.66) | Luspatercept | . |  |
| 0.07 (0.01 – 0.76) | 0.07 (0.01 – 0.45) | 0.08 (0.01 – 0.52) | 0.09 (0.01 – 0.51) | 0.09 (0.01 – 0.93) | 0.10 (0.01 – 0.67) | 0.14 (0.02 – 0.93) | 0.24 (0.05 – 1.13) | 0.56 (0.11 – 2.79) | ESA + G-CSF | . |
| 1.41 (0.28 – 7.18) | 1.45 (0.82 – 2.58) | 1.63 (0.87 – 3.06) | 1.66 (0.95 – 2.90) | 1.77 (0.27 – 11.76) | 1.92 (0.80 – 4.59) | 2.81 (1.35 – 5.89) | 4.73 (2.27 – 9.85) | 10.98 (5.62 – 21.47) | 19.51 (3.54 – 107.59) | Placebo |

### Table 5.2 Network Meta-analysis results for RBC–TI

The columns represent the comparison of the row drug class to the column drug class. The rows represent the comparison of the row drug class to the column drug class. The effect estimates are expressed as odds ratio and 95% confidence interval. Odds ratio <1 favors the drug in the column, Odds ratio >1 favors the drug in the row.

| Roxadustat | . | . | . | . | . |  |
| --- | --- | --- | --- | --- | --- | --- |
| 0.49 (0.18 – 1.35) | CC-486 | . | . | . | . |  |
| 0.47 (0.16 – 1.37) | 0.96 (0.33 – 2.83) | Imetelstat |  | . | . |  |
| 0.13 (0.03 – 0.62) | 0.25 (0.05 – 1.29) | 0.27 (0.05 – 1.38) | Lenalidomide |  |  |  |
| 0.11 (0.02 – 0.64) | 0.23 (0.04 – 1.31) | 0.24 (0.04 – 1.40) | 0.89 (0.45 – 1.75) | Lenalidomide +/- ESA +/- G-CSF | . | . |
| 0.06 (0.01 – 0.40) | 0.13 (0.02 – 0.82) | 0.13 (0.02 – 0.87) | 0.50 (0.20 – 1.24) | 0.57 (0.18 – 1.75) | Lenalidomide + ESA | . |
| 1.77 (0.88 – 3.57) | 3.60 (1.74 – 7.46) | 3.75 (1.69 – 8.34) | 14.15 (3.33 – 60.12) | 15.95 (3.23 – 78.80) | 28.17 (5.12 – 154.91) | Placebo |

### Table 5.3 Network Meta-analysis results for HI–P

The columns represent the comparison of the row drug class to the column drug class. The rows represent the comparison of the row drug class to the column drug class. The effect estimates are expressed as odds ratio and 95% confidence interval. Odds ratio <1 favors the drug in the column, Odds ratio >1 favors the drug in the row.

| Amifostine | . | . | . | . |  |
| --- | --- | --- | --- | --- | --- |
| 0.45 (0.03 – 6.23) | CC-486 | . | . | . |  |
| 0.25 (0.01 – 6.75) | 0.57 (0.06 – 5.78) | Decitabine | . | . |  |
| 0.14 (0.01 – 2.14) | 0.30 (0.07 – 1.35) | 0.54 (0.05 – 6.25) | Romiplostim | . |  |
| 0.08 (0.00 – 1.94) | 0.17 (0.02 – 1.62) | 0.30 (0.02 – 5.93) | 0.57 (0.05 – 6.11) | Eltrombopag |  |
| 2.10 (0.18 – 25.01) | 4.68 (1.93 – 11.32) | 8.24 (0.96 – 70.32) | 15.35 (4.65 – 50.69) | 27.10 (3.46 – 211.93) | Placebo |

### Table 5.4 Network Meta-analysis results for AEs

The columns represent the comparison of the row drug class to the column drug class. The rows represent the comparison of the row drug class to the column drug class. The effect estimates are expressed as odds ratio and 95% confidence interval. Odds ratio <1 favors the drug in the column, Odds ratio >1 favors the drug in the row.

| ESA | . |  | . |  |  |
| --- | --- | --- | --- | --- | --- |
| 0.29 (0.10 – 0.82) | Lenalidomide | . | . |  |  |
| 0.18 (0.01 – 4.26) | 0.64 (0.02 – 17.70) | Deferasirox + ESA | . | . | . |
| 0.11 (0.00 – 3.23) | 0.40 (0.02 – 10.42) | 0.63 (0.01 – 61.81) | CC-486 | . |  |
| 0.05 (0.02 – 0.13) | 0.18 (0.06 – 0.58) | 0.28 (0.01 – 7.49) | 0.45 (0.02 – 13.13) | Luspatercept |  |
| 0.34 (0.14 – 0.85) | 1.19 (0.69 – 2.05) | 1.87 (0.07 – 49.51) | 2.97 (0.12 – 73.78) | 6.59 (2.37 – 18.34) | Placebo |

### Table 5.5 Network Meta-analysis results for Dyspnoea

| ESA | . |  | . |  |
| --- | --- | --- | --- | --- |
| 0.62 (0.08 – 4.63) | Lenalidomide | . | . |  |
| 0.34 (0.14 – 0.85) | 0.55 (0.08 – 3.94) | Luspatercept | . |  |
| 0.20 (0.02 – 2.37) | 0.33 (0.02 – 5.36) | 0.60 (0.05 – 6.77) | Roxadustat |  |
| 1.26 (0.44 – 3.62) | 2.03 (0.37 – 11.17) | 3.70 (1.37 – 9.99) | 6.16 (0.68 – 56.09) | Placebo |

### Table 5.6 Network Meta-analysis results for SAEs

The columns represent the comparison of the row drug class to the column drug class. The rows represent the comparison of the row drug class to the column drug class. The effect estimates are expressed as odds ratio and 95% confidence interval. Odds ratio <1 favors the drug in the column, Odds ratio >1 favors the drug in the row.

| ESA | . |  | . | . |  |
| --- | --- | --- | --- | --- | --- |
| 1.00 (0.26 – 3.82) | Luspatercept | . | . | . |  |
| 0.90 (0.09 – 9.04) | 0.90 (0.06 – 13.01) | Deferasirox + ESA | . | . |  |
| 0.57 (0.15 – 2.15) | 0.57 (0.14 – 2.35) | 0.63 (0.04 – 9.03) | Romiplostim | . |  |
| 0.42 (0.11 – 1.64) | 0.42 (0.10 – 1.80) | 0.47 (0.03 – 6.82) | 0.75 (0.18 – 3.15) | Lenalidomide |  |
| 1.05 (0.43 – 2.54) | 1.05 (0.38 – 2.90) | 1.17 (0.10 – 13.80) | 1.86 (0.68 – 5.05) | 2.49 (0.89 – 7.01) | Placebo |

### Table 5.7 Network Meta-analysis results for Death

The columns represent the comparison of the row drug class to the column drug class. The rows represent the comparison of the row drug class to the column drug class. The effect estimates are expressed as odds ratio and 95% confidence interval. Odds ratio <1 favors the drug in the column, Odds ratio >1 favors the drug in the row.

| Luspatercept | . | . | . | . | . |  |
| --- | --- | --- | --- | --- | --- | --- |
| 0.65 (0.08 – 4.98) | Roxadustat | . | . | . | . |  |
| 0.63 (0.15 – 2.68) | 0.97 (0.19 – 4.96) | CC-486 | . | . | . |  |
| 0.41 (0.01 – 13.23) | 0.63 (0.02 – 22.12) | 0.65 (0.02 – 16.93) | Lenalidomide | . | . |  |
| 0.28 (0.02 – 3.76) | 0.43 (0.03 – 6.45) | 0.45 (0.05 – 4.42) | 0.69 (0.01 – 34.18) | ESA | . |  |
| 0.20 (0.01 – 6.54) | 0.31 (0.01 – 10.93) | 0.32 (0.01 – 8.38) | 0.49 (0.01 – 46.26) | 0.71 (0.01 – 35.63) | Decitabine |  |
| 0.61 (0.16 – 2.33) | 0.94 (0.20 – 4.37) | 0.97 (0.56 – 1.70) | 1.50 (0.06 – 37.12) | 2.17 (0.24 – 20.04) | 3.07 (0.12 – 77.69) | Placebo |

### Table 5.8 Network Meta-analysis results for Neutropenia

The columns represent the comparison of the row drug class to the column drug class. The rows represent the comparison of the row drug class to the column drug class. The effect estimates are expressed as odds ratio and 95% confidence interval. Odds ratio <1 favors the drug in the column, Odds ratio >1 favors the drug in the row.

| Roxadustat | . | . | . | . |  |
| --- | --- | --- | --- | --- | --- |
| 0.11 (0.00 – 3.53) | Lenalidomide + ESA | . | . |  |  |
| 0.10 (0.00 – 2.99) | 0.88 (0.53 – 1.44) | Lenalidomide | . | . |  |
| 0.06 (0.01 – 0.36) | 0.52 (0.02 –11.45) | 0.60 (0.03 – 12.55) | CC-486 | . |  |
| 0.01 (0.00 – 0.07) | 0.08 (0.00 – 1.95) | 0.09 (0.00 – 2.14) | 0.15 (0.04 – 0.54) | Imetelstat |  |
| 0.34 (0.06 – 1.91) | 3.10 (0.15 – 63.23) | 3.53 (0.18 – 69.25) | 5.92 (3.09 – 11.37) | 39.29 (13.16 – 117.34) | Placebo |

### Table 5.9 Network Meta-analysis results for Anaemia

The columns represent the comparison of the row drug class to the column drug class. The rows represent the comparison of the row drug class to the column drug class. The effect estimates are expressed as odds ratio and 95% confidence interval. Odds ratio <1 favors the drug in the column, Odds ratio >1 favors the drug in the row.

| Deferasirox + ESA | . | . | . | . | . |  |
| --- | --- | --- | --- | --- | --- | --- |
| 0.67 (0.09 – 4.99) | ESA | . | . | . | . |  |
| 0.31 (0.03 – 3.69) | 0.46 (0.11 – 1.98) | CC-486 | . | . | . |  |
| 0.15 (0.01 – 1.91) | 0.22 (0.04 – 1.09) | 0.47 (0.15 – 1.51) | Imetelstat | . | . |  |
| 0.13 (0.01 – 3.34) | 0.20 (0.02 – 2.48) | 0.43 (0.04 – 4.15) | 0.91 (0.09 – 9.73) | Lenalidomide | . |  |
| 0.06 (0.00 – 2.97) | 0.10 (0.00 – 2.51) | 0.21 (0.01 – 4.47) | 0.45 (0.02 – 10.22) | 0.49 (0.01 – 19.46) | Roxadustat |  |
| 0.33 (0.03 – 3.66) | 0.50 (0.14 – 1.83) | 1.08 (0.56 – 2.12) | 2.30 (0.88 – 5.97) | 2.52 (0.29 – 21.91) | 5.15 (0.26 – 101.65) | Placebo |

### Table 5.10 Network Meta-analysis results for Diarrhoea

The columns represent the comparison of the row drug class to the column drug class. The rows represent the comparison of the row drug class to the column drug class. The effect estimates are expressed as odds ratio and 95% confidence interval. Odds ratio <1 favors the drug in the column, Odds ratio >1 favors the drug in the row.

| Roxadustat | . | . | . | . | . | . | . |  |
| --- | --- | --- | --- | --- | --- | --- | --- | --- |
| 0.42 (0.10 – 1.81) | Imetelstat | . | . | . | . | . | . |  |
| 0.20 (0.05 – 0.83) | 0.47 (0.12 – 1.78) | ESA | . | . |  | . | . |  |
| 0.17 (0.05 – 0.59) | 0.40 (0.13 – 1.24) | 0.85 (0.28 – 2.58) | Lenalidomide | . | . |  | . |  |
| 0.16 (0.01 – 4.07) | 0.37 (0.01 – 9.27) | 0.80 (0.03 – 19.62) | 0.94 (0.04 – 21.41) | Eltrombopag | . | . | . |  |
| 0.13 (0.03 – 0.52) | 0.32 (0.09 – 1.12) | 0.68 (0.40 – 1.18) | 0.81 (0.29 – 2.21) | 0.86 (0.04 – 20.47) | Luspatercept | . | . |  |
| 0.09 (0.01 – 0.57) | 0.20 (0.03 – 1.26) | 0.44 (0.07 – 2.65) | 0.51 (0.12 – 2.13) | 0.55 (0.02 – 17.01) | 0.64 (0.11 – 3.65) | Lenalidomide + ESA | . |  |
| 0.06 (0.02 – 0.21) | 0.14 (0.05 – 0.44) | 0.30 (0.10 – 0.91) | 0.36 (0.15 – 0.84) | 0.38 (0.02 – 8.65) | 0.44 (0.16 – 1.21) | 0.69 (0.13 – 3.65) | CC-486 |  |
| 0.43 (0.14 – 1.28) | 1.02 (0.39 – 2.68) | 2.18 (0.86 – 5.51) | 2.57 (1.39 – 4.74) | 2.74 (0.13 – 58.85) | 3.19 (1.43 – 7.13) | 5.00 (1.06 – 23.48) | 7.21 (3.94 – 13.20) | Placebo |

### Table 5.11 Network Meta-analysis results for Nausea

The columns represent the comparison of the row drug class to the column drug class. The rows represent the comparison of the row drug class to the column drug class. The effect estimates are expressed as odds ratio and 95% confidence interval. Odds ratio <1 favors the drug in the column, Odds ratio >1 favors the drug in the row.

| Lenalidomide | . | . | . | . |  |
| --- | --- | --- | --- | --- | --- |
| 0.46 (0.12 – 1.80) | ESA | . |  | . | . |
| 0.34 (0.10 – 1.16) | 0.75 (0.17 – 3.27) | Roxadustat | . | . |  |
| 0.25 (0.08 – 0.85) | 0.56 (0.29 – 1.07) | 0.74 (0.20 – 2.77) | Luspatercept | . |  |
| 0.07 (0.03 – 0.20) | 0.16 (0.04 – 0.58) | 0.21 (0.07 – 0.65) | 0.28 (0.09 – 0.86) | CC-486 |  |
| 0.75 (0.35 – 1.64) | 1.65 (0.53 – 5.12) | 2.20 (0.86 – 5.64) | 2.96 (1.18 – 7.46) | 10.47 (5.59 – 19.62) | Placebo |

### Table 5.12 Network Meta-analysis results for Constipation

The columns represent the comparison of the row drug class to the column drug class. The rows represent the comparison of the row drug class to the column drug class. The effect estimates are expressed as odds ratio and 95% confidence interval. Odds ratio <1 favors the drug in the column, Odds ratio >1 favors the drug in the row.

| Imetelstat | . | . | . | . | . | . |  |
| --- | --- | --- | --- | --- | --- | --- | --- |
| 0.51 (0.13 – 2.04) | Luspatercept | . | . | . | . | . |  |
| 0.39 (0.01 – 11.52) | 0.76 (0.03 – 21.96) | Eltrombopag | . | . | . | . |  |
| 0.28 (0.08 – 1.02) | 0.55 (0.16 – 1.84) | 0.72 (0.03 – 19.87) | Lenalidomide | . | . | . |  |
| 0.19 (0.06 – 0.64) | 0.38 (0.13 – 1.15) | 0.50 (0.02 – 13.36) | 0.70 (0.26 – 1.87) | CC-486 | . | . |  |
| 0.08 (0.00 – 1.83) | 0.17 (0.01 – 3.48) | 0.22 (0.00 – 16.66) | 0.30 (0.02 – 6.12) | 0.43 (0.02 – 8.36) | ESA | . |  |
| 0.07 (0.01 – 0.72) | 0.14 (0.01 – 1.36) | 0.18 (0.00 – 8.51) | 0.26 (0.03 – 2.36) | 0.37 (0.04 – 3.16) | 0.84 (0.02 – 29.81) | Roxadustat |  |
| 0.63 (0.22 – 1.77) | 1.23 (0.49 – 3.11) | 1.62 (0.06 – 40.83) | 2.26 (1.03 – 4.96) | 3.23 (1.79 – 5.82) | 7.44 (0.41 – 135.14) | 8.83 (1.11 – 70.45) | Placebo |

### Table 5.13 Network Meta-analysis results for Fatigue

The columns represent the comparison of the row drug class to the column drug class. The rows represent the comparison of the row drug class to the column drug class. The effect estimates are expressed as odds ratio and 95% confidence interval. Odds ratio <1 favors the drug in the column, Odds ratio >1 favors the drug in the row.

| CC-486 | . | . | . | . | . |  |
| --- | --- | --- | --- | --- | --- | --- |
| 0.77 (0.31 – 1.97) | ESA | . | . |  | . |  |
| 0.56 (0.17 – 1.87) | 0.72 (0.21 – 2.41) | Roxadustat | . | . | . |  |
| 0.48 (0.17 – 1.34) | 0.62 (0.22 – 1.73) | 0.86 (0.24 – 3.10) | Lenalidomide | . |  |  |
| 0.35 (0.14 – 0.87) | 0.45 (0.26 – 0.80) | 0.63 (0.19 – 2.07) | 0.73 (0.27 – 2.00) | Luspatercept | . |  |
| 0.22 (0.05 – 0.97) | 0.28 (0.06 – 1.26) | 0.39 (0.07 – 2.10) | 0.45 (0.15 – 1.35) | 0.62 (0.14 – 2.72) | Lenalidomide + ESA | . |
| 1.08 (0.56 – 2.09) | 1.40 (0.72 – 2.70) | 1.94 (0.70 – 5.35) | 2.26 (1.03 – 4.96) | 3.08 (1.66 – 5.74) | 4.97 (1.30 – 19.06) | Placebo |

## Appendix 6. Certainty of the effect estimates

### Table 6.1 Certainty of the effect estimates for HI–E

| Comparison | Within-study bias | Reporting bias | Indirectness | Imprecision | Heterogeneity | Incoherence | Confidence rating |
| --- | --- | --- | --- | --- | --- | --- | --- |
| Amifostine: Placebo | No concerns | Low risk | No concerns | Some concerns | No concerns | No concerns | Low |
| CC-486: Placebo | No concerns | Low risk | No concerns | Some concerns | Some concerns | No concerns | Low |
| Deferasirox + ESA: ESA | No concerns | Low risk | No concerns | Some concerns | No concerns | No concerns | Low |
| ESA: ESA + G-CSF | No concerns | Low risk | No concerns | Some concerns | Some concerns | No concerns | Low |
| ESA: Luspatercept | No concerns | Low risk | No concerns | No concerns | Some concerns | No concerns | Moderate |
| ESA: Placebo | No concerns | Low risk | No concerns | No concerns | Some concerns | No concerns | Moderate |
| Imetelstat: Placebo | No concerns | Low risk | No concerns | Some concerns | Some concerns | No concerns | Low |
| Lenalidomide: Lenalidomide +/-ESA+/-G-CSF | No concerns | Low risk | No concerns | Some concerns | No concerns | No concerns | Low |
| Lenalidomide: Lenalidomide + ESA | No concerns | Low risk | No concerns | No concerns | Major concerns | No concerns | Low |
| Lenalidomide: Placebo | No concerns | Low risk | Some concerns | Some concerns | Some concerns | No concerns | Low |
| Luspatercept: Placebo | No concerns | Low risk | No concerns | No concerns | No concerns | No concerns | High |
| Amifostine: CC-486 | No concerns | Low risk | No concerns | Some concerns | No concerns | No concerns | Low |
| Amifostine: Deferasirox + ESA | No concerns | Low risk | No concerns | Some concerns | No concerns | No concerns | Low |
| Amifostine: ESA | No concerns | Low risk | No concerns | Major concerns | No concerns | No concerns | Low |
| Amifostine: ESA + G-CSF | No concerns | Low risk | No concerns | No concerns | Major concerns | No concerns | Low |
| Amifostine: Imetelstat | No concerns | Low risk | No concerns | Major concerns | No concerns | No concerns | Low |
| Amifostine: Lenalidomide | No concerns | Low risk | Some concerns | Major concerns | No concerns | No concerns | Low |
| Amifostine: Lenalidomide +/- ESA +/- G-CSF | No concerns | Low risk | No concerns | Major concerns | No concerns | No concerns | Low |
| Amifostine: Lenalidomide + ESA | No concerns | Low risk | No concerns | Major concerns | No concerns | No concerns | Low |
| Amifostine: Luspatercept | No concerns | Low risk | No concerns | No concerns | Major concerns | No concerns | Low |
| CC-486: Deferasirox + ESA | No concerns | Low risk | No concerns | Major concerns | No concerns | No concerns | Low |
| CC-486: ESA | No concerns | Low risk | No concerns | No concerns | Major concerns | No concerns | Low |
| CC-486: ESA + G-CSF | No concerns | Low risk | No concerns | No concerns | Major concerns | No concerns | Low |
| CC-486: Imetelstat | No concerns | Low risk | No concerns | Major concerns | No concerns | No concerns | Low |
| CC-486: Lenalidomide | No concerns | Low risk | Some concerns | Major concerns | No concerns | No concerns | Low |
| CC-486: Lenalidomide +/- ESA +/- G-CSF | No concerns | Low risk | No concerns | Major concerns | No concerns | No concerns | Low |
| CC-486: Lenalidomide + ESA | No concerns | Low risk | No concerns | Major concerns | No concerns | No concerns | Low |
| CC-486: Luspatercept | No concerns | Low risk | No concerns | No concerns | Some concerns | No concerns | Moderate |
| Deferasirox+ESA: ESA + G-CSF | No concerns | Low risk | No concerns | No concerns | Major concerns | No concerns | Low |
| Deferasirox + ESA: Imetelstat | No concerns | Low risk | No concerns | Major concerns | No concerns | No concerns | Low |
| Deferasirox + ESA: Lenalidomide | No concerns | Low risk | No concerns | Major concerns | No concerns | No concerns | Low |
| Deferasirox + ESA: Lenalidomide +/-ESA+/-G-CSF | No concerns | Low risk | No concerns | Major concerns | No concerns | No concerns | Low |
| Deferasirox + ESA: Lenalidomide + ESA | No concerns | Low risk | No concerns | Major concerns | No concerns | No concerns | Low |
| Deferasirox + ESA: Luspatercept | No concerns | Low risk | No concerns | No concerns | Major concerns | No concerns | Low |
| Deferasirox + ESA: Placebo | No concerns | Low risk | No concerns | Major concerns | No concerns | No concerns | Low |
| ESA: Imetelstat | No concerns | Low risk | No concerns | No concerns | Major concerns | No concerns | Low |
| ESA: Lenalidomide | No concerns | Low risk | Some concerns | No concerns | Major concerns | No concerns | Low |
| ESA: Lenalidomide +/- ESA +/- G-CSF | No concerns | Low risk | No concerns | Major concerns | No concerns | No concerns | Low |
| ESA:Lenalidomide + ESA | No concerns | Low risk | No concerns | Major concerns | No concerns | No concerns | Low |
| ESA + G-CSF: Imetelstat | No concerns | Low risk | No concerns | No concerns | Major concerns | No concerns | Low |
| ESA + G-CSF: Lenalidomide | No concerns | Low risk | No concerns | No concerns | Major concerns | No concerns | Low |
| ESA + G-CSF: Lenalidomide +/-ESA +/- G-CSF | No concerns | Low risk | No concerns | No concerns | Major concerns | No concerns | Low |
| ESA + G-CSF: Lenalidomide + ESA | No concerns | Low risk | No concerns | No concerns | Major concerns | No concerns | Low |
| ESA + G-CSF: Luspatercept | No concerns | Low risk | No concerns | Major concerns | No concerns | No concerns | Low |
| ESA + G-CSF: Placebo | No concerns | Low risk | No concerns | No concerns | Major concerns | No concerns | Low |
| Imetelstat: Lenalidomide | No concerns | Low risk | Some concerns | Major concerns | No concerns | No concerns | Low |
| Imetelstat: Lenalidomide +/- ESA +/-G-CSF | No concerns | Low risk | No concerns | Major concerns | No concerns | No concerns | Low |
| Imetelstat: Lenalidomide + ESA | No concerns | Low risk | No concerns | Major concerns | No concerns | No concerns | Low |
| Imetelstat: Luspatercept | No concerns | Low risk | No concerns | No concerns | Some concerns | No concerns | Moderate |
| Lenalidomide: Luspatercept | No concerns | Low risk | Some concerns | No concerns | No concerns | No concerns | Moderate |
| Lenalidomide + ESA: Lenalidomide +/- ESA +/- G-CSF | No concerns | Low risk | No concerns | Major concerns | No concerns | No concerns | Low |
| Lenalidomide +/- ESA +/- G-CSF: Luspatercept | No concerns | Low risk | No concerns | No concerns | Major concerns | No concerns | Low |
| Lenalidomide +/- ESA +/- G-CSF: Placebo | No concerns | Low risk | Some concerns | Some concerns | Some concerns | No concerns | Low |
| Lenalidomide + ESA: Luspatercept | No concerns | Low risk | No concerns | No concerns | Major concerns | No concerns | Low |
| Lenalidomide + ESA: Placebo | No concerns | Low risk | Some concerns | No concerns | Major concerns | No concerns | Low |

### Table 6.2. Certainty of the effect estimates for RBC–TI

| Comparison | Within-study bias | Reporting bias | Indirectness | Imprecision | Heterogeneity | Incoherence | Confidence rating |
| --- | --- | --- | --- | --- | --- | --- | --- |
| CC-486: Placebo | No concerns | Low risk | No concerns | No concerns | Major concerns | No concerns | Low |
| Imetelstat: Placebo | No concerns | Low risk | No concerns | No concerns | Major concerns | No concerns | Low |
| Lenalidomide: Lenalidomide +/-ESA +/- G-CSF | No concerns | Low risk | No concerns | Major concerns | No concerns | No concerns | Low |
| Lenalidomide: Lenalidomide + ESA | No concerns | Low risk | No concerns | Some concerns | Some concerns | No concerns | Moderate |
| Lenalidomide: Placebo | No concerns | Low risk | No concerns | No concerns | Major concerns | No concerns | Low |
| Placebo: Roxadustat | No concerns | Low risk | No concerns | Some concerns | Some concerns | No concerns | Low |
| CC-486: Imetelstat | No concerns | Low risk | No concerns | Major concerns | No concerns | No concerns | Low |
| CC-486: Lenalidomide | No concerns | Low risk | No concerns | Major concerns | No concerns | No concerns | Low |
| CC-486: Lenalidomide +/- ESA +/- G-CSF | No concerns | Low risk | No concerns | Major concerns | No concerns | No concerns | Low |
| CC-486: Lenalidomide + ESA | No concerns | Low risk | No concerns | No concerns | Major concerns | No concerns | Low |
| CC-486: Roxadustat | No concerns | Low risk | No concerns | Major concerns | No concerns | No concerns | Low |
| Imetelstat: Lenalidomide | No concerns | Low risk | No concerns | Major concerns | No concerns | No concerns | Low |
| Imetelstat: Lenalidomide +/- ESA +/-G-CSF | No concerns | Low risk | No concerns | Major concerns | No concerns | No concerns | Low |
| Imetelstat: Lenalidomide + ESA | No concerns | Low risk | No concerns | No concerns | Major concerns | No concerns | Low |
| Imetelstat: Roxadustat | No concerns | Low risk | No concerns | Major concerns | No concerns | No concerns | Low |
| Lenalidomide: Roxadustat | No concerns | Low risk | No concerns | No concerns | Major concerns | No concerns | Low |
| Lenalidomide + ESA: Lenalidomide +/- ESA +/- G-CSF | No concerns | Low risk | No concerns | Major concerns | No concerns | No concerns | Low |
| Lenalidomide +/- ESA +/- G-CSF: Placebo | No concerns | Low risk | No concerns | No concerns | Major concerns | No concerns | Low |
| Lenalidomide +/- ESA +/- G-CSF: Roxadustat | No concerns | Low risk | No concerns | No concerns | Major concerns | No concerns | Low |
| Lenalidomide + ESA: Placebo | No concerns | Low risk | No concerns | No concerns | Major concerns | No concerns | Low |
| Lenalidomide + ESA: Roxadustat | No concerns | Low risk | No concerns | No concerns | Major concerns | No concerns | Low |

### Table 6.3. Certainty of the effect estimates for HI–P

| Comparison | Within-study bias | Reporting bias | Indirectness | Imprecision | Heterogeneity | Incoherence | Confidence rating |
| --- | --- | --- | --- | --- | --- | --- | --- |
| Amifostine: Placebo | No concerns | Low risk | No concerns | Major concerns | No concerns | Major concerns | Low |
| CC-486: Placebo | No concerns | Low risk | No concerns | No concerns | Major concerns | Major concerns | Low |
| Decitabine: Placebo | No concerns | Low risk | No concerns | Some concerns | Some concerns | Major concerns | Low |
| Eltrombopag: Placebo | No concerns | Low risk | No concerns | No concerns | Major concerns | Major concerns | Low |
| Placebo: Romiplostim | No concerns | Low risk | No concerns | No concerns | Major concerns | Major concerns | Low |
| Amifostine: CC-486 | No concerns | Low risk | No concerns | Major concerns | No concerns | Major concerns | Low |
| Amifostine: Decitabine | No concerns | Low risk | No concerns | Major concerns | No concerns | Major concerns | Low |
| Amifostine: Eltrombopag | No concerns | Low risk | No concerns | Major concerns | No concerns | Major concerns | Low |
| Amifostine: Romiplostim | No concerns | Low risk | No concerns | Major concerns | No concerns | Major concerns | Low |
| CC-486: Decitabine | No concerns | Low risk | No concerns | Major concerns | No concerns | Major concerns | Low |
| CC-486: Eltrombopag | No concerns | Low risk | No concerns | Major concerns | No concerns | Major concerns | Low |
| CC-486: Romiplostim | No concerns | Low risk | No concerns | Major concerns | No concerns | Major concerns | Low |
| Decitabine: Eltrombopag | No concerns | Low risk | No concerns | Major concerns | No concerns | Major concerns | Low |
| Decitabine: Romiplostim | No concerns | Low risk | No concerns | Major concerns | No concerns | Major concerns | Low |
| Eltrombopag: Romiplostim | No concerns | Low risk | No concerns | Major concerns | No concerns | Major concerns | Low |

### Table 6.4. Certainty of the effect estimates for AEs

| Comparison | Within-study bias | Reporting bias | Indirectness | Imprecision | Heterogeneity | Incoherence | Confidence rating |
| --- | --- | --- | --- | --- | --- | --- | --- |
| CC-486: Placebo | No concerns | Low risk | No concerns | Major concerns | No concerns | No concerns | Low |
| ESA: Placebo | No concerns | Low risk | No concerns | No concerns | Major concerns | No concerns | Low |
| Lenalidomide: Placebo | No concerns | Low risk | No concerns | Major concerns | No concerns | No concerns | Low |
| Luspatercept: Placebo | No concerns | Low risk | No concerns | No concerns | Major concerns | No concerns | Low |
| Deferasirox + ESA: Placebo | No concerns | Low risk | No concerns | Major concerns | No concerns | No concerns | Low |

## Appendix 7. Treatment ranking (P-scores)

### Table 7.1 Treatment ranking (P-scores) for efficacy outcomes

| **HI–E** | | **RBC–TI** | | **HI–P** | |
| --- | --- | --- | --- | --- | --- |
| ESA + G-CSF | 0.9648 | Lenalidomide + ESA | 0.8850 | Eltrombopag | 0.8683 |
| Luspatercept | 0.9202 | CC-486 | 0.7934 | Romiplostim | 0.7747 |
| ESA | 0.7549 | Lenalidomide +/- ESA +/- G-CSF | 0.6818 | Decitabine | 0.5951 |
| Lenalidomide + ESA | 0.619 | Lenalidomide | 0.6129 | CC-486 | 0.4323 |
| Lenalidomide +/- ESA +/- G-CSF | 0.4347 | Imetelstat | 0.3437 | Amifostine | 0.2683 |
| Deferasirox+ESA | 0.3911 | Roxadustat | 0.1740 | Placebo | 0.0614 |
| CC-486 | 0.3707 | Placebo | 0.0092 |  |  |
| Imetelstat | 0.3598 |  |  |  |  |
| Lenalidomide | 0.3146 |  |  |  |  |
| Amifostine | 0.2807 |  |  |  |  |
| Placebo | 0.0896 |  |  |  |  |

### Table 7.2 Treatment ranking (P-scores) for safety outcomes

| **AEs** | **Dyspnoea** | **SAEs** | | **Death** | **Fatigue** |
| --- | --- | --- | --- | --- | --- |
| ESA 0.9466 | Placebo 0.8503 | Placebo 0.6958 | | Luspatercept 0.7512 | Placebo 0.8838 |
| Placebo 0.6279 | ESA 0.7244 | ESA 0.6369 | | Roxadustat 0.5760 | CC-486 0.8040 |
| Lenalidomide 0.5168 | Lenalidomide 0.5096 | Luspatercept 0.6299 | | CC-486 0.5665 | ESA 0.6545 |
| Deferasirox + ESA 0.4497 | Luspatercept 0.2375 | Deferasirox + ESA 0.5458 | | Placebo 0.5442 | Roxadustat 0.4670 |
| CC-486 0.3491 | Roxadustat 0.1782 | Romiplostim 0.3106 | | Lenalidomide 0.4504 | Lenalidomide 0.3901 |
| Luspatercept 0.1098 |  | Lenalidomide 0.1810 | | ESA 0.3211 | Luspatercept 0.2075 |
|  |  |  | | Decitabine 0.2905 | Lenalidomide+ESA 0.0931 |
|  |  |  | |  | |
| **Neutropenia** | **Anaemia** | **Diarrhoea** | | **Nausea** | **Constipation** |
| Roxadustat 0.9386 | Deferasirox + ESA 0.8386 | Roxadustat 0.9570 | | Lenalidomide 0.9152 | Imetelstat 0.8928 |
| Placebo 0.7351 | ESA 0.8057 | Placebo 0.7809 | | Placebo 0.7966 | Placebo 0.7635 |
| Lenalidomide + ESA 0.5271 | Placebo 0.5899 | Imetelstat 0.7580 | | ESA 0.5860 | Luspatercept 0.6695 |
| Lenalidomide 0.4313 | CC-486 0.5393 | ESA 0.5097 | | Roxadustat 0.4227 | Eltrombopag 0.5592 |
| CC-486 0.3419 | Lenalidomide 0.2947 | Eltrombopag 0.4368 | | Luspatercept 0.2756 | Lenalidomide 0.4375 |
| Imetelstat 0.0261 | Imetelstat 0.2456 | Lenalidomide 0.4296 | | CC-486 0.0039 | CC-486 0.3079 |
|  | Roxadustat 0.1863 | Luspatercept 0.3206 |  | | ESA 0.2228 |
|  |  | Lenalidomide + ESA 0.2216 |  | | Roxadustat 0.1468 |
|  |  | CC-486 0.0859 |  | |  |

## Appendix 8. Forest plot of node-splitting analysis

### Figure 8.1 Forest plot of node-splitting analysis for HI–E


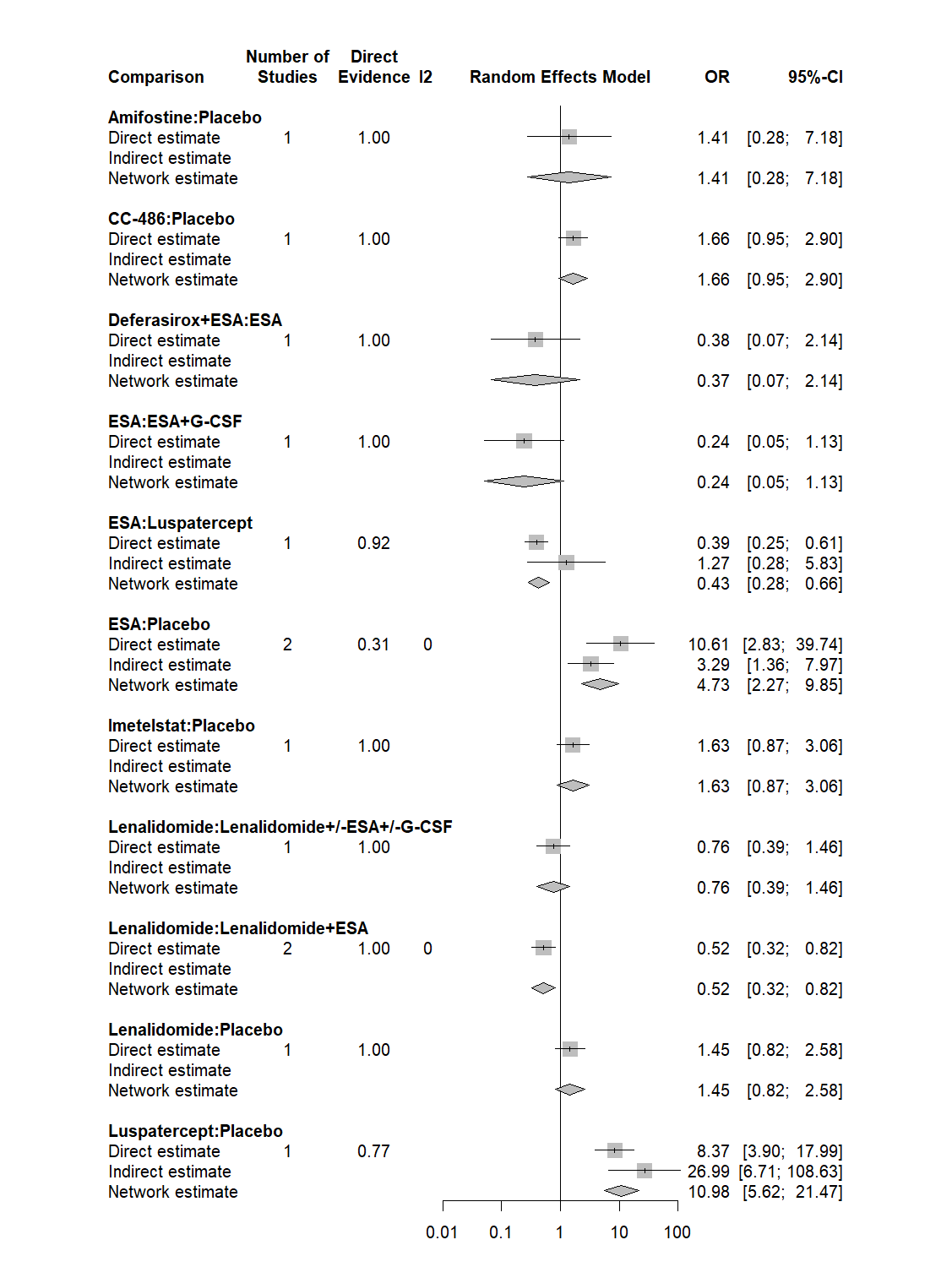


### Figure 8.2 Forest plot of node-splitting analysis for HI–P


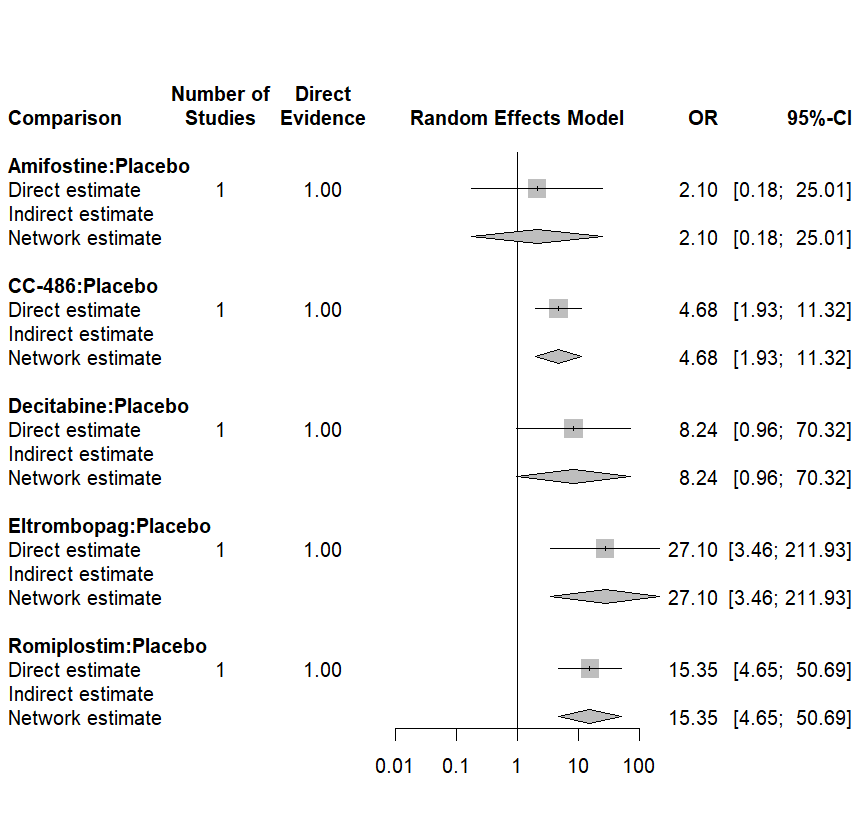


### Figure 8.3 Forest plot of node-splitting analysis for AEs


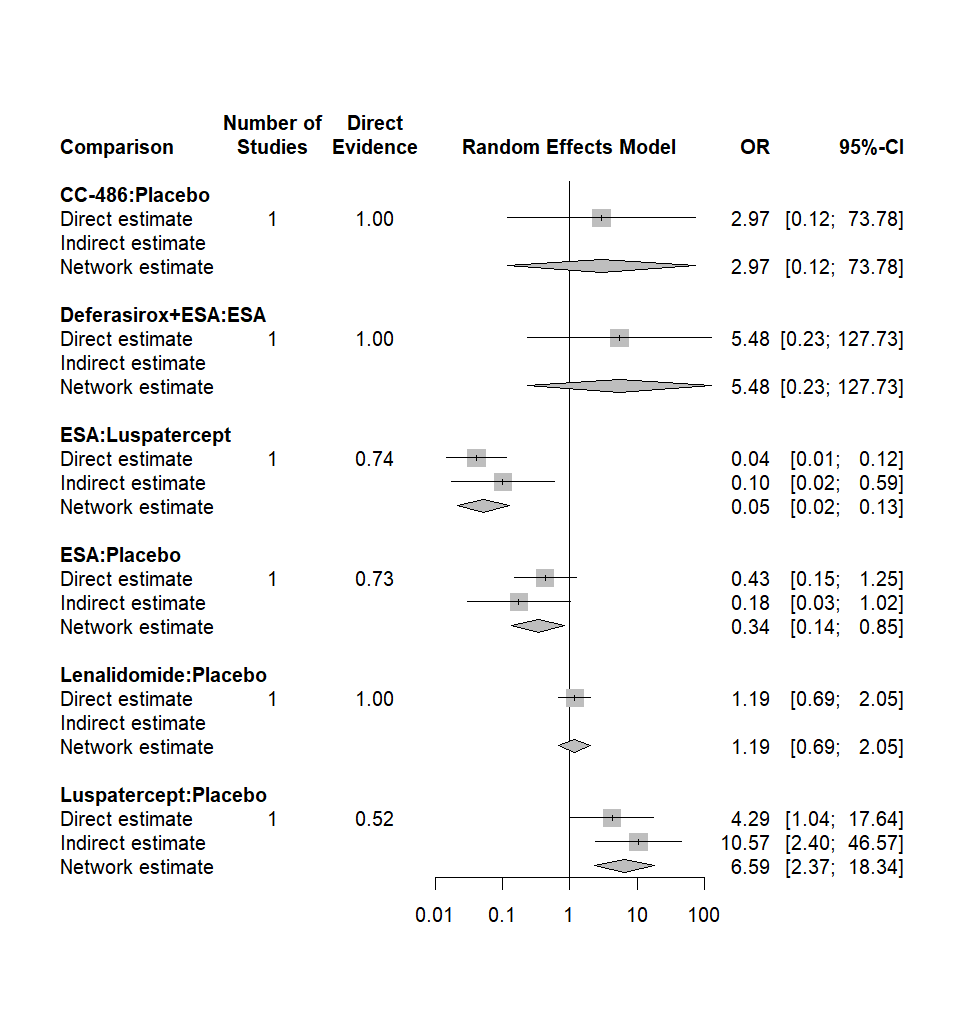


## Appendix 9. Sensitivity analysis

### Table 9.1. Sensitivity analysis for RBC–TI after excluding the IWG-2018 Trial

| Treatment | Main Analysis  OR (95% CI) | Main analysis P score | Sensitivity Analysis  OR (95% CI) | Sensitivity analysis P score |
| --- | --- | --- | --- | --- |
| Lenalidomide + ESA | 28.17 (5.12 – 154.91) | 0.8850 | 28.17 (5.12 – 154.91) | 0.8623 |
| CC-486 | 3.60 (1.74 – 7.46) | 0.7934 | 3.60 (1.74 – 7.46) | 0.7522 |
| Lenalidomide +/- ESA +/- G-CSF | 15.95 (3.23 – 78.80) | 0.6818 | 15.95 (3.23 – 78.80) | 0.6195 |
| Lenalidomide | 14.15 (3.33 – 60.12) | 0.6129 | 14.15 (3.33 – 60.12) | 0.5366 |
| Imetelstat | 3.75 (1.69 – 8.34) | 0.3437 | 3.75 (1.69 – 8.34) | 0.2291 |
| Placebo |  | 0.0092 |  | 0.0002 |

### Table 9.2. Sensitivity analysis for HI–E after excluding high risk of bias trials

| Treatment | Main analysis  OR (95% CI) | Main analysis P score | Sensitivity analysis  OR (95% CI) | Sensitivity analysis P score |
| --- | --- | --- | --- | --- |
| ESA + G CSF | 19.51 (3.54 – 107.59) | 0.9648 | 20.41 (3.52 – 118.51) | 0.9539 |
| Luspatercept | 10.98 (5.62 – 21.47) | 0.9202 | 11.15 (5.46 – 22.78) | 0.8850 |
| ESA | 4.73 (2.27 – 9.85) | 0.7549 | 4.95 (2.24 – 10.92) | 0.6819 |
| Deferasirox + ESA | 1.77 (0.27 – 11.76) | 0.3911 | 1.86 (0.27 – 12.89) | 0.3713 |
| CC 486 | 1.66 (0.95 – 2.90) | 0.3707 | 1.66 (0.88 – 3.13) | 0.3588 |
| Imetelstat | 1.63 (0.87 – 3.06) | 0.3598 | 1.63 (0.81 – 3.28) | 0.3484 |
| Amifostine | 1.41 (0.28 – 7.18) | 0.2807 | 1.41 (0.27 – 7.38) | 0.2932 |
| Placebo | – | 0.0896 | – | 0.1074 |

### Table 9.3. Sensitivity analysis for RBC–TI after excluding high risk of bias trials

| Treatment | Main analysis  OR (95% CI) | Main analysis P‑score | Sensitivity analysis  OR (95% CI) | Sensitivity analysis P‑score |
| --- | --- | --- | --- | --- |
| CC‑486 | 3.60 (1.74 – 7.46) | 0.7934 | 3.60 (1.74 – 7.46) | 0.9949 |
| Imetelstat | 3.75 (1.69 – 8.34) | 0.3437 | 3.75 (1.69 – 8.34) | 0.6436 |
| Roxadustat | 1.77 (0.88 – 3.57) | 0.1740 | 1.77 (0.88 – 3.57) | 0.3432 |
| Placebo | – | 0.0092 | – | 0.0183 |

## Appendix 10. Baseline patient characteristics

| Study | Patients,  n | Randomised treatments,  n | Age | Male,  n(%) | Serum EPO,  mU/mL | WHO classification  (%) |
| --- | --- | --- | --- | --- | --- | --- |
| Balleari2006 | 30 | ESA:15 | 74(59-89) ^a^ | 9(60.0%) | 300(35-810) ^c^ | RA:27%; RARS:20%; RCMD:20%; RAEB:20%; 5q:13%  RA:40%; RARS:13%; RCMD:27%; RAEB:13%; 5q:7% |
|  |  | ESA + G-CSF:15 | 75(62-87) ^a^ | 10(66.7%) | 354(47-754) ^c^ |  |
| Toma2016 | 131 | Lenalidomide:65 | 73(64-77) ^b^ | 40(61.5%) | 134(69-306) ^b^ | RARS:40%; RCMD:32%; RAEB:11%; MDS-U:17%  RARS:49%; RCMD:25%; RAEB:23%; MDS-U:3% |
|  |  | Lenalidomide+ ESA:65 | 74(68-76) ^b^ | 48(72.7%) | 185(73-499) ^b^ |  |
| Thepot2016 | 98 | Azacitidine:49 | 72(67-78) ^c^ | 34(69.4%) | – | RA:4%; RARS:49%; RCMD:28%; RAEB:10%; CMML:6%  RA:8%; RARS:35%; RCMD:34%; RAEB:14%; CMML:8% |
|  |  | Azacitidine + ESA:49 | 73(62-78) ^c^ | 34(69.4%) |  |  |
| Platzbecker2017 | 146 | Placebo:49 | 73(66-80) ^b^ | 29(59.2%) | – | RA:27%; RARS:8%; RCMD:39%; RAEB:20%; 5q:4%; MDS-U:2%  RA:9%; RARS:18%; RCMD:46%; RAEB:13%; 5q:11%; MDS-U:1% |
|  |  | ESA:97 | 74(68-79) ^b^ | 51(52.6%) |  |  |
| Fenaux2018 | 130 | Placebo:45 | 75(36-87) ^c^ | 25(55.6%) | – | RA:25%; RARS:5%; RCMD:58%; RAEB:2%; 5q:7%  RA:8%; RARS:11%; RCMD:57%; RAEB:13%; 5q:2%; MDS-U:1% |
|  |  | ESA:85 | 85(40-94) ^c^ | 46(54.1%) |  |  |
| Gattermann2018 | 23 | Deferasirox + ESA:11 | 71(58-83) ^c^ | – | – |  |
|  |  | ESA:12 | 76(60-81) ^c^ |  |  |  |
| Loosdrecht2024 | 184 | Lenalidomide:92 | 71(41-84) ^c^ | 51(55.4%) | 248(15-968) ^c^ | RA:2%; RARS:14%; RCMD:53%; RAEB:10%; 5q:14%; MDS-U:3%  RARS:16%; RCMD:44%; RAEB:15%; 5q:18%; MDS-U:1% |
|  |  | Lenalidomide+/-ESA+/-G-CSF:92 | 73(38-89) ^c^ | 49(53.3%) | 192(7-859) ^c^ |  |
| Porta2024 | 363 | Luspatercept:182 | 74(68-80) ^c^ | 109(59.9%) | – |  |
|  |  | ESA:181 | 74(69-80) ^c^ | 92(50.8%) |  |  |
| A.F. List2021 | 195 | Lenalidomide + ESA:99 | 73(47-92) ^c^ | 74(74.7%) | 151(19-3,264) ^c^ | RA:7%; RARS:29%; RCMD:40%; RAEB:1%; MDS-U:16%; CMML:2%  RA:6%; RARS:25%; RCMD:41%; RAEB:11%; MDS-U:12%; CMML:4% |
|  |  | Lenalidomide:96 | 74(49-89) ^c^ | 65(67.7%) | 160(11-3,048) ^c^ |  |
| Garcia-Manero2018 | 239 | Placebo:79 | 70(43-87) ^c^ | 54(68.4%) | – |  |
|  |  | Lenalidomide:160 | 71(46-87) ^c^ | 108(67.5%) |  |  |
| Garcia-Manero2021 | 216 | Placebo:109 | 73(44-88) ^c^ | 79(72.5%) | – | RA:3%; RARS:2%; RCMD:67%; RAEB:27%; MDS-U:1%  RA:4%; RARS:3%; RCMD:75%; RAEB:16%; MDS-U:2% |
|  |  | CC-486:107 | 74(30-89) ^c^ | 79(73.8%) |  |  |
| Ye2021 | 82 | Placebo:41 | 61(52-69) ^b^ | – | – | RARS:5%; RCMD:56%; RAEB:32%; RCUD:7%  RARS:2%; RCMD:32%; RAEB:49%; RCUD:17% |
|  |  | Decitabine:41 | 59(44-68) ^b^ | – |  |  |
| Fenaux2020 | 229 | Placebo:76 | 72(26-91) ^c^ | 50(65.8%) | – | – |
|  |  | Luspatercept:153 | 71(40-95) ^c^ | 94(61.4%) |  |  |
| Platzbecker2024 | 178 | Placebo:60 | 73(69-78) ^c^ | 40(66.7%) | 277(72-621) ^b^ | – |
|  |  | Imetelstat:118 | 72(65-75) ^c^ | 71(60.2%) | 175(77-455) ^b^ |  |
| Mittelman2024 | 140 | Placebo:58 | 71(65-77) ^b^ | 37(63.8%) | 117(56-283) ^b^ | – |
|  |  | Roxadustat:82 | 72(67-80) ^b^ | 46(56.1%) | 94(54-257) ^b^ |  |
| Giagounidis2014 | 250 | Placebo:83 | 69(61-76) ^b^ | 53(63.9%) | – | RA:6%; RCMD:69%; RAEB:11%; MDS-U:14%  RA:4%; RARS:1%; RCMD:70%; RAEB:15%; MDS-U:10% |
|  |  | Romiplostim:167 | 71(62-77) ^b^ | 95(56.9%) |  |  |
| Oliva2017 | 90 | Placebo:31 | 67 ± 15 ^d^ | 15(48.4%) | – | – |
|  |  | Eltrombopag:59 | 70 ± 11 ^d^ | 37(62.7%) |  |  |
| Schanz2009 | 44 | Placebo:22 | 68 ± 8 ^d^ | 12(54.5%) | – | RA:45%; RARS:36%; RAEB:9%; CMML:10%  RA:55%; RARS:41%; RAEB:4% |
|  |  | Amifostine:22 | 66 ± 10 ^d^ | 15(68.2%) |  |  |
| Ghaderi2020 | 27 | β-D mannuronic acid + vitamin B12 + Folic acid:13 | 59 ± 4 ^d^ | 7(53.8%) | – | – |
|  |  | Vitamin B12 + Folic acid:14 | 59 ± 3 ^d^ | 7(50.0%) |  |  |
| Yang2020 | 82 | PND + Andriosl Testocaps + Thalidomide:41 | 54 ± 11 ^d^ | 12(29.3%) | – | – |
|  |  | Andriol Testocaps + Thalidomide;41 | 53 ± 14 ^d^ | 14(34.1%) |  |  |

Note: Data are presented as: a mean(range); b median(IQR); c median(range); d mean ± SD.

“–” = endpoint not assessed or not reported.

## Appendix 11. Summary of endpoint definitions and response criteria

| Study | Criteria | Primary endpoint | Endpoint | Duration | Definition |
| --- | --- | --- | --- | --- | --- |
| Balleari2006 | IWG-2006 | HI–E | HI–E | ≥8 weeks | – |
| Toma2016 | IWG-2006 | HI–E | HI–E; RBC–TI | ≥8 weeks | HI–E was defined as reduction of RBC transfusions of at least 4 units during the 8 weeks prior to evaluation compared to the pretreatment period, for transfusions performed at hemoglobin levels < 9 g/dl. |
| Thepot2016 | IWG-2006 | RBC–TI | HI–E; RBC–TI | ≥8 weeks | RBC–TI was defined as no need for red blood cell transfusions (performed at a Hb level of less than 9g/dl), with a stable hemoglobin level ≥ 9g/dl lasting for at least 8 weeks. |
| Platzbecker2017 | IWG-2006 | HI–E | HI–E | ≥8 weeks | HI–E was defined as ≥ 1.5 g/dl increase from baseline in hemoglobin with a mean rise of ≥ 1.5 g/dl for 8 weeks. |
| Fenaux2018 | IWG-2006 | HI–E | HI–E | ≥8 weeks | HI–E was defined by (1) patients with an increase in Hb level by at least 1.5 g/dL lasting less than 8 weeks due to epoetin-α discontinuation were considered responders if, when restarting epoetin-α at lower dose, Hb still increased by at least 1.5 g/dL, (2) in transfused patients, the baseline Hb value was taken before the last transfusion preceding enrollment (rather than after), and (3) if discrepancies were observed between local and centralized Hb levels, the latter should be used to evaluate response and its duration. |
| Gattermann2018 | IWG-2006 | HI–E | HI–E;  HI–P | ≥8 weeks | HI–E was defined as increase from baseline in Hb ≥ 1.5g/dL. Red blood cell transfusion independence lasting at least 12 weeks with a concurrent mean haemoglobin increase of at least 1·5 g/dL (weeks 1–24). HI–P was defined as platelets <100×10^9^/L at baseline and increase of ≥ 30×10^9^/L. |
| Loosdrecht2024 | IWG-2006 | HI–E | HI–E; RBC–TI | ≥8 weeks | – |
| Porta2024 | IWG-2006 | RBC–TI | HI–E | ≥8 weeks | – |
| A.F. List2021 | IWG-2006 | HI–E | HI–E | ≥8 weeks | HI–E was defined by (1) achievement of RBC transfusion independence for ≥ 8 consecutive weeks accompanied by a sustained ≥ 1 g/dL hemoglobin rise compared with mean pretransfusion baseline value in transfusion-dependent patients (≥ 4 U RBC/8 week) or (2) a > 2 g/dL rise in hemoglobin without transfusion for ≥ 8 consecutive weeks in nontransfusion-dependent patients (< 4 U RBC/8 week). |
| Garcia-Manero2018 | IWG-2006 | RBC–TI | HI–E; TI | ≥8 weeks | – |
| Garcia-Manero2021 | IWG-2006 | RBC–TI | HI–E; RBC–TI; HI–P | ≥8 weeks | – |
| Ye2021 | IWG-2006 | HI | HI–P | ≥8 weeks | – |
| Fenaux2020 | IWG-2006 | HI–E | HI–E | ≥8 weeks | – |
| Platzbecker2024 | IWG-2006 | RBC–TI | HI–E; RBC–TI | ≥8 weeks | – |
| Mittelman2024 | IWG-2018 | RBC–TI | TI | ≥8 weeks | – |
| Giagounidis2014 | IWG-2006 | HI–P | HI–P | ≥8 weeks | – |
| Oliva2017 | IWG-2006 | HI–P | HI–P | ≥8 weeks | – |
| Schanz2009 | IWG-2006 | HI | HI–E; HI–P | ≥8 weeks | – |
| Ghaderi2020 | IWG-2006 | HI | HI–E; HI–P | ≥8 weeks | – |
| Yang2020 | IWG-2006 | HI | HI–P | ≥8 weeks | – |

## Appendix 12. Baseline characteristics and effect modifier distribution across treatment nodes

| Treatment | Trials,  (n) | Baseline EPO  (mU/mL),  Median(range) | TD patients, (%) | RS-positive, (%) | IPSS Low/Int-1, (%) |
| --- | --- | --- | --- | --- | --- |
| ESA | 5 | 73.5 (35.8–810) * | 0-100 | 72 (only Porta2024); others – | 99–100 |
| ESA + G CSF | 1 | 354 (47–754) | – | – | 100 |
| Lenalidomide | 4 | 160 (134–248) ‡ | 100 | – | 100 |
| Lenalidomide + ESA | 2 | 168 (151–185) | 100 | – | 98-100 |
| Lenalidomide+/-ESA+/-G CSF | 1 | 192 (7–859) | 100 | – | 98 |
| Azacitidine | 1 | – | 100 | – | 98 |
| Azacitidine + ESA | 1 | – | 100 | – | 100 |
| Deferasirox + ESA | 1 | – | – | – | – |
| Luspatercept | 2 | – | 100 | 73 | 99-100# |
| CC 486 | 1 | – | 100 | – | 99 |
| Decitabine | 1 | – | – | – | 95 |
| Imetelstat | 1 | 175 (77–455) | 100 | 62 | 100 |
| Roxadustat | 1 | 94 (54–257) | 100 | – | 100# |
| Romiplostim | 1 | – | – | – | 96 |
| Eltrombopag | 1 | – | – | – | 100 |
| Amifostine | 1 | – | – | – | 100 |
| β-D mannuronic acid + vitamin B12 + Folic acid | 1 | – | – | – | 100 |
| Vitamin B12 + folic acid | 1 | – | – | – | 100 |

Abbreviations: TD, transfusion dependent; RS, ring sideroblast; IPSS, International Prognostic Scoring System; Int-1, intermediate-1.

Note: – endpoint not assessed or not reported.

#Trial used IPSS‑R: very low + low + intermediate.

‡From Toma2016 (134), List2021 (160), Loosdrecht2024 (248).

*Based on three reported values: Balleari2006 (300), Platzbecker2017 (73.5), Porta2024.

## Appendix 13. Absolute risk differences and number needed to treat

.

| Outcome | Placebo rate | Treatment | OR (95% CI) | ARD (95% CI) | NNT (95% CI) |
| --- | --- | --- | --- | --- | --- |
| RBC–TI | 10.5% | Lenalidomide + ESA | 28.17 (5.12 – 154.91) | 74.0% (34.4% – 95.1%) | 1.4 (1.1 – 2.9) |
|  |  | Lenalidomide +/- ESA +/- G-CSF | 15.95 (3.23 – 78.80) | 61.1% (18.3% – 88.7%) | 1.6 (1.1 – 5.5) |
|  |  | Lenalidomide | 14.15 (3.33 – 60.12) | 58.0% (18.9% – 85.6%) | 1.7 (1.2 – 5.3) |
|  |  | Imetelstat | 3.75 (1.69 – 8.34) | 22.4% (6.5% – 43.0%) | 4.5 (2.3 – 15.4) |
|  |  | CC‑486 | 3.60 (1.74 – 7.46) | 21.4% (7.1% – 40.4%) | 4.7 (2.5 – 14.1) |
|  |  | Roxadustat | 1.77 (0.88 – 3.57) | 7.5% (–1.2% – 20.8%) | NA |
| HI–P | 4.5% | Eltrombopag | 27.10 (3.46 – 211.93) | 51.9% (9.5 – 86.4) | 1.9 (1.2 – 10.5) |
|  |  | Romiplostim | 15.35 (4.65 – 50.69) | 37.6% (13.5 – 66.0) | 2.7 (1.5 – 7.4) |
|  |  | CC‑486 | 4.68 (1.93 – 11.32) | 13.7% (3.8 – 30.3) | 7.3 (3.3 – 26.3) |
|  |  | Decitabine | 8.24 (0.96 – 70.32) | 23.7% (–0.2% – 72.3%) | NA |
|  |  | Amifostine | 2.10 (0.18 – 25.01) | 4.6% (–3.7% – 49.6%) | NA |
| HI–E | 23.4% | ESA + G-CSF | 19.51 (3.54 – 107.59) | 62.3% (24.1 – 92.8) | 1.6 (1.1 – 4.1) |
|  |  | Luspatercept | 10.98 (5.62 – 21.47) | 53.6% (34.5 – 63.3) | 1.9 (1.6 – 2.9) |
|  |  | ESA | 4.73 (2.27 – 9.85) | 35.7% (15.0 – 53.2) | 2.8 (1.9 – 6.7) |
|  |  | Lenalidomide + ESA | 2.81 (1.35 – 5.89) | 22.7% (4.8 – 39.5) | 4.4 (2.5 – 20.8) |

Abbreviations: ARD, absolute risk difference; NNT, number needed to treat

Note: NA because the 95% confidence interval of the odds ratio includes 1, indicating no statistically significant difference from placebo.

## Appendix 14. Hypothesis‑generating clinical framework for treatment selection in LR‑MDS

| Patient subgroup | Considered therapies | Guideline position  (NCCN / ESMO) | Notes |
| --- | --- | --- | --- |
| Anaemia,  non‑del(5q),  EPO ≤ 200 mU/mL ¹ | ESA +/- G‑CSF | First line | HI‑E benefit clear;  RBC‑TI evidence weaker |
| Anaemia,  ESA‑failure,  RS‑positive | Luspatercept | Recommended for ESA‑failure, RS‑positive | Monitor fatigue, dyspnoea |
| Anaemia,  ESA‑failure,  transfusion dependent,  non‑del(5q),  RS‑negative | Lenalidomide +/- ESA;  CC‑486;  Imetelstat | NCCN provides category specific recommendations for Imetelstat ² | Monitor myelosuppression (neutropenia, gastrointestinal toxicity) with CC‑486/Imetelstat |
| Del(5q) ±  transfusion dependent | Lenalidomide | NCCN: Category 1;  EMSO: Standard of care | Monitor cytopenias, thrombocytopenia |
| Thrombocytopenia | Eltrombopag/Romiplostim | TPO‑RAs | Limited trial evidence |

¹ NCCN guidelines typically use EPO ≤500 mU/mL as a reference threshold for ESA responsiveness; we use a more conservative ≤200 mU/mL to align with the lower EPO levels in ESA trial populations (median 73.5 mU/mL).

² NCCN guidelines provide specific recommendations for imetelstat based on EPO level and RS status.
